# Modelling historical changes in the force-of-infection of Chagas disease to inform control and elimination programmes: application in Colombia

**DOI:** 10.1136/bmjgh-2017-000345

**Published:** 2017-09-07

**Authors:** Zulma M Cucunubá, Pierre Nouvellet, Lesong Conteh, Mauricio Javier Vera, Victor Manuel Angulo, Juan Carlos Dib, Gabriel Jaime Parra -Henao, María Gloria Basáñez

**Affiliations:** 1 Department of Infectious Disease Epidemiology, Faculty of Medicine (St Mary's campus), Imperial College London, London, UK; 2 Department of Infectious Disease Epidemiology, London Centre for Neglected Tropical Disease Research (LCNTDR), Imperial College London, London, UK; 3 Grupode Parasitología—RED CHAGAS, Instituto Nacional de Salud, Bogotá, Colombia; 4 Department of Infectious Disease Epidemiology, Faculty of Medicine (St Mary's campus), Medical Research Council Centre for Outbreak Analysis and Modelling, School of Public Health, Imperial College London, London, UK; 5 Department of Infectious Disease Epidemiology, Faculty of Medicine (St Mary's campus), Health Economics Group, School of Public Health, Imperial College London, London, UK; 6 Grupo de Enfermedades Endemo-Epidémicas, Subdirección Enfermedades Transmisibles, Ministerio de Salud y Protección Social, Bogotá, Colombia; 7 Centro de Investigaciones en Enfermedades Tropicales (CINTROP), Universidad Industrial de Santander, Piedecuesta, Colombia; 8 Fundación Salud para el Trópico, SantaMarta, Colombia; 9 Instituto Colombiano de Medicina Tropical, Universidad CES, Santa Martha, Colombia

**Keywords:** chagas disease, serology, mathematical modelling, cross-sectional survey, control strategies

## Abstract

**Background:**

WHO's 2020 milestones for Chagas disease include having all endemic Latin American countries certified with no intradomiciliary *Trypanosoma cruzi* transmission, and infected patients under care. Evaluating the variation in historical exposure to infection is crucial for assessing progress and for understanding the priorities to achieve these milestones.

**Methods:**

Focusing on Colombia, all the available age-structured serological surveys (undertaken between 1995 and 2014) were searched and compiled. A total of 109 serosurveys were found, comprising 83 742 individuals from rural (indigenous and non-indigenous) and urban settings in 14 (out of 32) administrative units (departments). Estimates of the force-of-infection (FoI) were obtained by fitting and comparing three catalytic models using Bayesian methods to reconstruct temporal and spatial patterns over the course of three decades (between 1984 and 2014).

**Results:**

Significant downward changes in the FoI were identified over the course of the three decades, and in some specific locations the predicted current seroprevalence in children aged 0–5 years is <1%. However, pronounced heterogeneity exists within departments, especially between indigenous, rural and urban settings, with the former exhibiting the highest FoI (up to 66 new infections/1000 people susceptible/year). The FoI in most of the indigenous settings remain unchanged during the three decades investigated. Current prevalence in adults in these 15 departments varies between 10% and 90% depending on the dynamics of historical exposure.

**Conclusions:**

Assessing progress towards the control of Chagas disease requires quantifying the impact of historical exposure on current age-specific prevalence at subnational level. In Colombia, despite the evident progress, there is a marked heterogeneity indicating that in some areas the vector control interventions have not been effective, hindering the possibility of achieving interruption by 2020. A substantial burden of chronic cases remains even in locations where serological criteria for transmission interruption may have been achieved, therefore still demanding diagnosis and treatment interventions.

**Key questions**

**What is already known about this topic?**

WHO’s milestones for 2020 include elimination of intradomiciliary (vectorial) transmission of Chagas disease in endemic Latin American countries as well as provision of care to acute and chronically infected individuals. We sought to evaluate progress towards these aims illustrating our approach with Colombia. We did not identify recent studies modelling spatial and temporal trends in Chagas disease incidence (measured by the force-of-infection (FoI)) at national or subnational scale with the purpose of addressing the question of whether Colombia is on track to achieve the 2020 goals.

**What are the new findings?**

We find that in Colombia, the FoI has decreased in many locations probably due to interventions undertaken in the last three decades, but substantial heterogeneity remains at subnational and epidemiological setting levels. The FoI in Amerindian (indigenous) populations has remained essentially unchanged and can be 60 times as high as in urban settings, suggesting that no effective interventions have been implemented in the former. These heterogeneities indicate that achieving transmission interruption by 2020 is unlikely. In addition to this, even in areas with a marked reduction in incidence, we find a large remnant of prevalent cases in the adult population (exposed to higher FoI earlier in life), indicating that this age group bears most of the disease burden and is/will be in need of treatment and/or care.

**Recommendations for policy**

We argue that FoI models fitted to seroprevalence data such as those applied here could be used to analyse retrospectively the many serosurveys already available in Latin America. This would allow quantification of incidence trends and determination of age-specific prevalence for assessment of intradomiciliary transmission interruption. Greater efforts should be made in the region in terms of vector control and particularly in ensuring a systematic data collection of both surveillance and vector control activities to corroborate the finding from seroprevalence studies. This would greatly assist efforts towards Chagas disease prevention, control and care in the region.

## Introduction

Chagas disease is of great public health concern in the American continent, where an estimated 5.7 million people are affected,^1^ and 271 000–1 054 000 disability-adjusted life years are lost mainly due to heart complications and premature deaths.^2 3^ In 2012, WHO set milestones for Chagas disease, namely, achieving interruption of transmission via blood transfusion by 2015 and interruption of intradomiciliary vectorborne transmission by 2020,^4^ as well as having all infected/ill people under care.^5^ In addition to the verification of absence of triatomines in a specific area with a well-conducted surveillance programme, reaching a seroprevalence <2% among children aged under 5 years has been set as an operational threshold to indicate transmission interruption.^6^ However challenging, quantifying whether endemic countries are on track to achieve the 2020 goals is, therefore, timely and imperative.

Control strategies for Chagas disease have mostly focused on insecticide-spraying campaigns against triatomine vectors and screening of transfusion units in blood banks. Countries of the Southern Cone have achieved a substantial decrease in prevalence; Brazil, Chile and Uruguay have successfully interrupted transmission by *Triatoma infestans*, the principal vector in the subregion, as defined by the Pan American Health Organization. The Andean Countries Initiative (Iniciativa de Países Andinos (IPA)) started in 1997 in Colombia, Ecuador, Peru and Venezuela. In Colombia, IPA aimed to control intradomiciliary transmission by *Rhodnius prolixus* and introduced mandatory screening of blood banks. Due to greater vector diversity in the Andean subregion and administrative difficulties in implementing vector control, progress has been slow, and interruption of vectorial transmission has yet to be achieved or demonstrated.^7^ Accurate description of temporal and spatial patterns of incidence and prevalence is a prerequisite for the design of optimal strategies for interruption of transmission, diagnosis of prevalent cases and treatment as well as for monitoring and evaluation of progress made towards national and regional goals.

In Colombia, an estimated 437 960 people are infected and 131 388 live with chronic cardiomyopathy,^1^ and <1% have access to diagnosis and aetiological treatment.^8^ Therefore, even if vectorial transmission were interrupted, past exposure must be assessed to design and implement appropriate diagnostic and treatment strategies. Taking Colombia as a case study, this paper aims to characterise past and present epidemiological trends of Chagas disease based on historical seroprevalence data. The key parameter of interest in this study is the ‘per susceptible’ (at-risk) rate of infection acquisition, more commonly referred to as the ‘force-of-infection’ (FoI). A better understanding of FoI patterns will enable identification of (i) active transmission areas, (ii) areas where past control strategies have succeeded (or failed) and (iii) local variations in disease burden. Using data compiled on an unprecedented scale, we reconstruct and identify trends in spatial and temporal transmission of Chagas disease in Colombia. The paper aims to ascertain heterogeneity in incidence and prevalence and to present a quantitative framework to address the question of whether Colombia in particular is on track to achieve the 2020 goals for Chagas disease set by WHO. We argue that, more generally, our approach could be applied to the analysis of seroprevalence data from other countries in Latin America to assist endemic countries’ efforts in assessing Chagas disease control progress and needs in the region.

## Methods

### Survey selection and data extraction

PubMed, Web of Knowledge and the Latin American Index (Literatura Latinoamericana de Información en Ciencias de la Salud, in Spanish) databases were searched for the period 1995–2014, with no year or language restrictions. Non-published data and grey literature including personal collections, research degree theses filed in Colombian universities and unpublished reports from control programmes at the National Health Institute and Ministry of Health in Colombia were also scrutinised.

Surveys had to meet all of the following inclusion criteria: (i) be population based (not hospital based), (ii) specify individuals’ age or age group, (iii) indicate diagnostic test(s) used and (iv) identify location and date of sample collection. When detailed information was not available, corresponding authors were contacted. If the reports contained several surveys conducted in different locations or years, each survey was extracted as a unit. Settings were categorised (according to the National Statistics Department, Colombia) as rural (indigenous or non-indigenous), or urban. Indigenous settings comprised those with Amerindian populations mostly following traditional lifestyles (Arhuaco, Bari, Hitnú, Kogui, Uwa and Wiwa communities).^9^ Seroprevalence data for those aged <1 year were excluded to avoid biases due to passive antibody transfer from mother to fetus. Each serosurvey was cross-validated to eliminate duplication of records. Details of the literature search are described in online supplementary data (text A) and online supplementary table 1 .

10.1136/bmjgh-2017-000345.supp1 Supplementary file 1

### Data analysis, catalytic models and FoI estimation

Descriptive prevalence results are reported as percentages and accompanied by 95% binomial (exact―Clopper-Pearson) CIs (95% CI). For the FoI models, we consider that if the rate of infection acquisition―here the rate of seroconversion―is constant over time, infection (sero)prevalence will increase monotonically with age as cumulative exposure increases. Formally, $P_{a}=1-exp\left(-\lambda a\right)$, with $P_{a}$ the age-specific seroprevalence and λ the FoI (the per susceptible incidence) as originally described by Muench.^10 11^ More generally, the FoI may fluctuate over time τ, modifying the seroprevalence age profiles. For a survey completed at time τ, $P_{a,\tau }=1-exp\left(-\int_{t=\tau -a}^{t=\tau }\lambda_{\;t}dt\right)$.^12^ Therefore, a serosurvey completed at time τ, and including ages a from $\left\{a_{min},a_{max}\right\}$, is informative on exposure (and FoI) between $\left\{\tau ,a_{max}\right\}$ and τ. Other modelling assumptions included: (a) no age-dependency in transmission,^12^ (b) no seroreversion^12^ and (c) no specific migration due to Chagas infection status.^12 13^

Based on this, three catalytic models were compared:

*Model 1:* a model with constant FoI ($\lambda_{t}=\lambda$), as described above.^11^

(1) $$P_{a}=1-exp\left(-\lambda \;a\right)$$

*Model 2:* a model with discrete change(s) in FoI. If a survey is conducted at time τ, and it is assumed that the FoI was constant ($\lambda_{1}$) previous to time $\xi_{1}$, but subsequently modified (to $\lambda_{2}$), for example, due to an intervention, remaining constant thereafter, the expected seroprevalence for an individual born before $\xi_{1}$ would be,

(2) $$P_{a,\tau }=1-exp\left(-\left[\lambda_{1}\left(a-\left(\tau -\xi_{1}\right)\right)+\lambda_{2}\left(\tau -\xi_{1}\right)\right]\right)$$

and the expected seroprevalence for an individual born after $\xi_{1}$ would be $P_{a,\tau }=1-exp\left(-\lambda_{2}\;a\right)$. In this case, $\xi_{1}$ is also a parameter to be estimated. We considered up to four discrete changes in FoI (for estimation of up to five values of $\lambda {\;}_{i}$ and up to four values of $\xi_{i}$).

*Model 3:* a model with yearly variation in the FoI,

(3) $$P_{a,\tau }=1-exp\left(-\sum_{i=\tau -a+1}^{i=\tau }\lambda {}_{i}\right)$$

First, each model (1–3 above) was fitted to data from each of the 109 serosurveys to estimate the FoI and understand how best to explain incidence and prevalence patterns. Second, the same models were fitted to data aggregated at subnational (first administrative, department-specific) level. As the Sierra Nevada de Santa Marta (SNSM) covers an extensive area (21 158 km^2^) belonging to three departments (Cesar, Guajira and Magdalena), yet encompassing unique epidemiological conditions not typical of the component departments,^14^ it was considered as a separate area for the subnational analysis. Third, setting-specific FoI was examined, allowing separate analyses to be conducted according to rural indigenous, rural non-indigenous, urban and mixed settings.

### Model fitting and comparison

We formulated a likelihood for each model containing the expected (predicted) prevalence according to equations 1–3 above (parameter dependent), assuming that observations follow a binomial distribution (text B of online supplementary data). For all three models, expected prevalence was adjusted to account for the sensitivity and specificity of the diagnostic test(s) used in each seroprevalence study (text C of online supplementary data). The parameters’ joint posterior distribution was obtained using Bayesian Markov Chain Monte Carlo sampling with the Metropolis-Hastings Algorithm,^15^ implemented in R (V.3.2.2),^16^ assuming uninformative (flat) priors for all parameters and performing 1 00 000 iterations. After discarding 20 000 initial burn-in iterations and thinning to retain 1 in every 50 samples, the median and 95% Bayesian credible intervals (95% BCI) of the parameters were obtained. The deviance information criterion (DIC) was used to compare the models, with a difference >10 units taken to indicate a significant difference in the goodness of fit to the data.^17^ When models had similar DIC values, the most parsimonious model (the one with the least number of parameters) was preferred. Spatial correlation between the values of FoI derived from the serological surveys (using model 3) was investigated by fitting semivariograms using the geoR package^18^ (see text D of online supplementary data).

### Ethics

This study was approved by the Ethics Committee of the National Institute of Health in Colombia (Code CTIN N° 14–2011) under the principles embodied in the Declaration of Helsinki.

## Results

**Figure 1:**
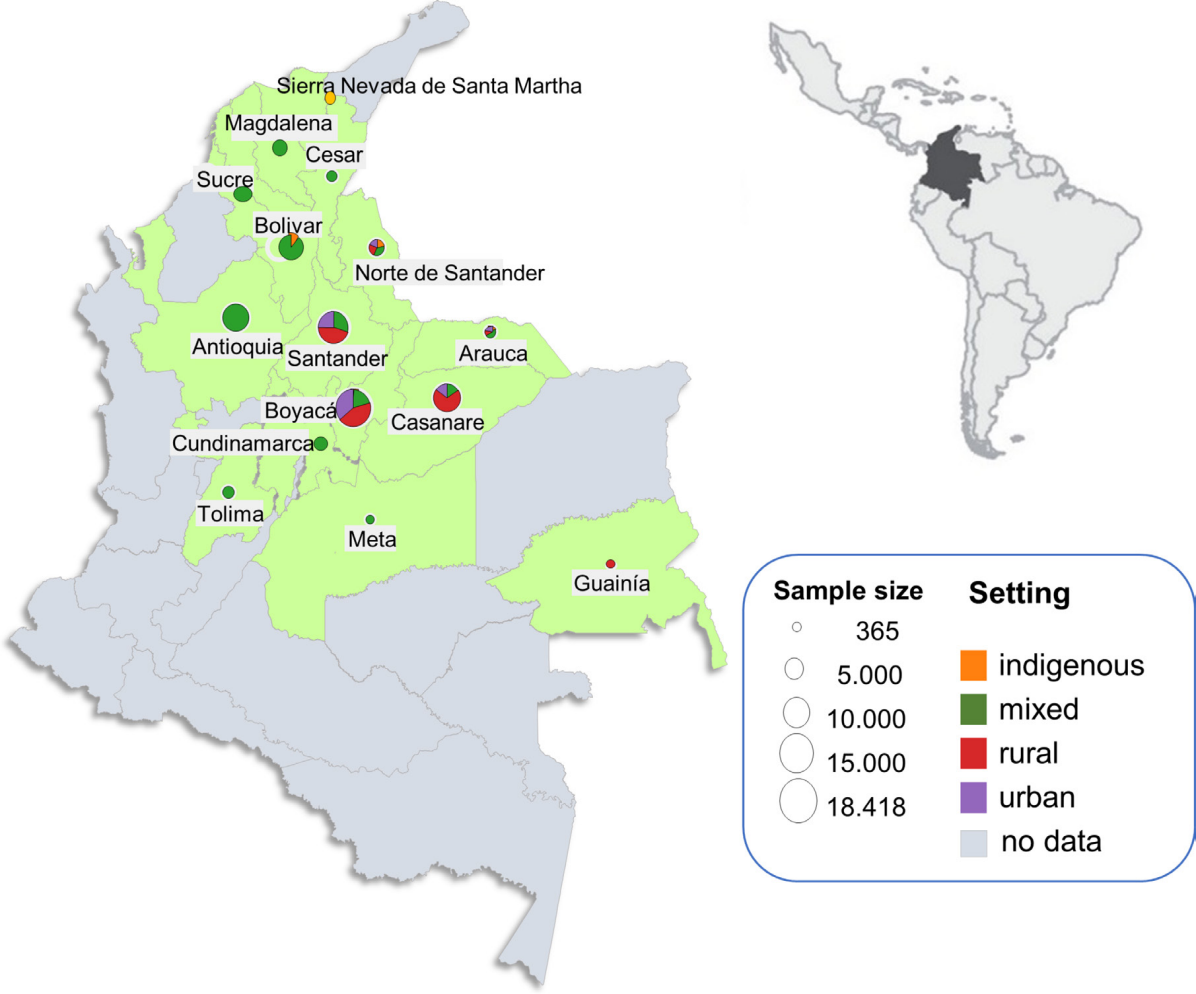
Geographical distribution of serosurveys of *Trypanosoma cruzi* infection in Colombia. Locations are endemic departments (in green); size of the circles represents sample size; colours represent type of epidemiological setting; for departments in grey there are no available seroprevalence data. Inset shows location of Colombia in Latin America.

The compiled dataset comprised 83 742 individuals from 15 endemic departments who participated in 109 serosurveys from 1995 to 2014 (see online supplementary table 1). From those, seven serosurveys (in four communities) were of rural indigenous populations, 46 of rural non-indigenous, 33 of urban and 23 of mixed locations (see online supplementary figure 1). The geographical distribution of the serosurveys is presented in figure 1. Prevalence of Chagas infection varied markedly across departments, settings and age groups. The highest prevalence values were found among the indigenous communities (Arhuacos, Koguis and Wiwas) of the SNSM in 2000 (47.1%; 95% CI 43.1% to 54.3%) and the Hitnú community in 2012 (48.7%; 95% CI 42.6% to 51.6%). The lowest prevalence values were found in urban settings of Boyacá and Santander (see online supplementary table 1). All the FoI values given in the following sections are expressed per 1000 at-risk (susceptible) population per year.

### FoI from individual serosurveys

**Figure 2:**
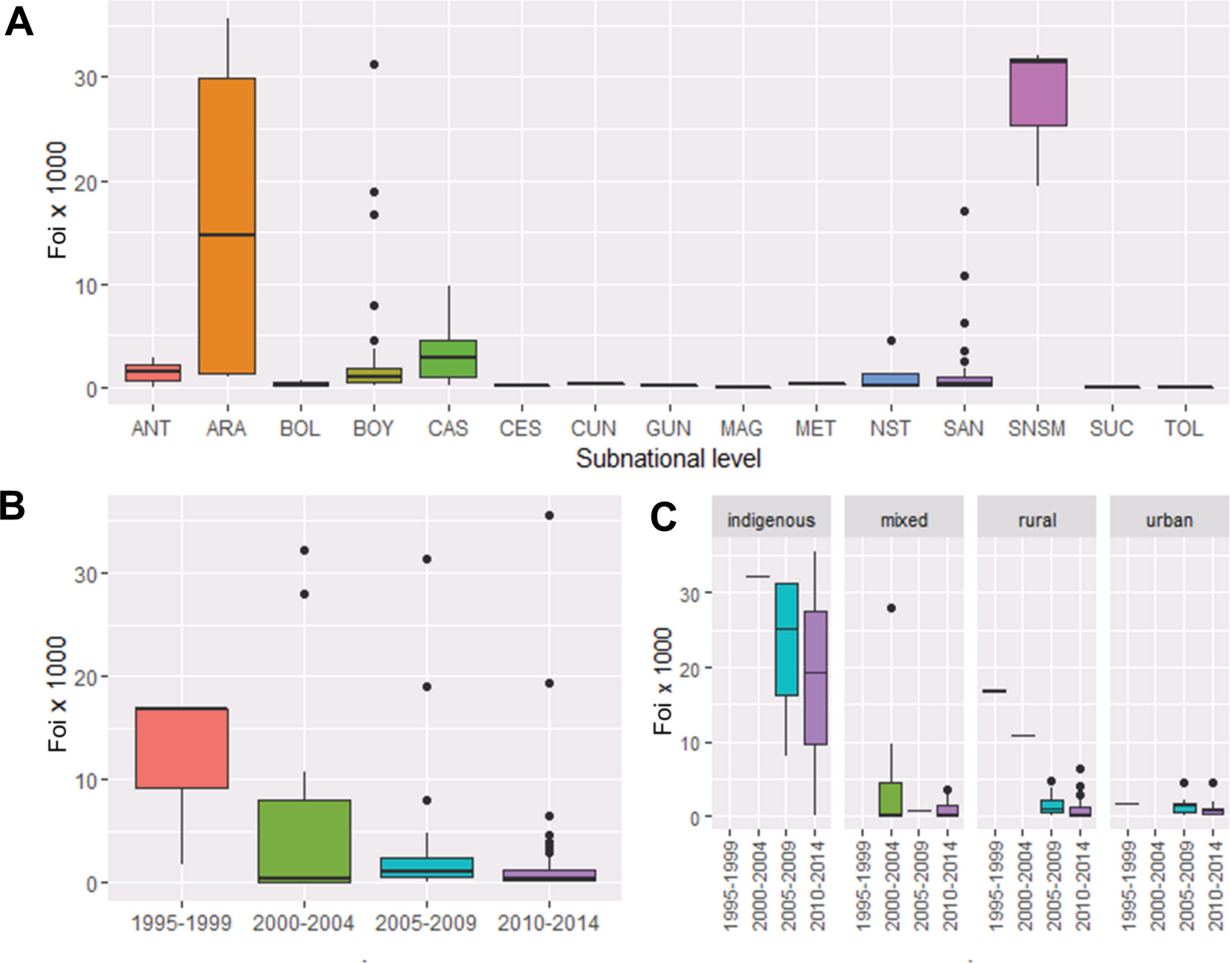
Geographical, temporal and setting-level heterogeneities in the force-of-infection (FoI) of Chagas disease in Colombia. (A) Averaged FoI from individual serosurveys for subnational (first administrative) level (departments) per 1000 population at-risk per year. ANT, Antioquia; ARA, Arauca; BOL, Bolívar; CAS, Casanare; CES, Cesar; CUN, Cundinamarca; GUN, Guainía; MAG, Magdalena; MET, Meta; NST, Norte Santander; SAN, Santander; SNSM, Sierra Nevada de Santa Marta; SUC, Sucre; TOL, Tolima. The location of the departments has been provided in figure 1. (B) Averaged (overall) FoI by quinquennial period. (C) Averaged FoI by quinquennial period and type of setting (rural indigenous, mixed, rural-non-indigenous and urban). Bottom and top of the box are first and third quartiles; the horizontal band inside is the median; the whiskers are the minimum and maximum 1.5 IQR and the solid circles are the outlier values.

The best-fit models for each individual serosurvey are presented in online supplementary table 2. A marked heterogeneity in the FoI was observed both temporally and geographically (figure 2 and online supplementary figure 2). The FoI was observed to vary between (subnational) administrative departments (figure 2A) and overall to decline with time (figure 2B) and from indigeneous to urban settings (figure 2C). The spatial analysis showed non-significant spatial correlation between the FoI values derived from individual serosurveys (see online supplementary figure 3).

**Figure 3:**
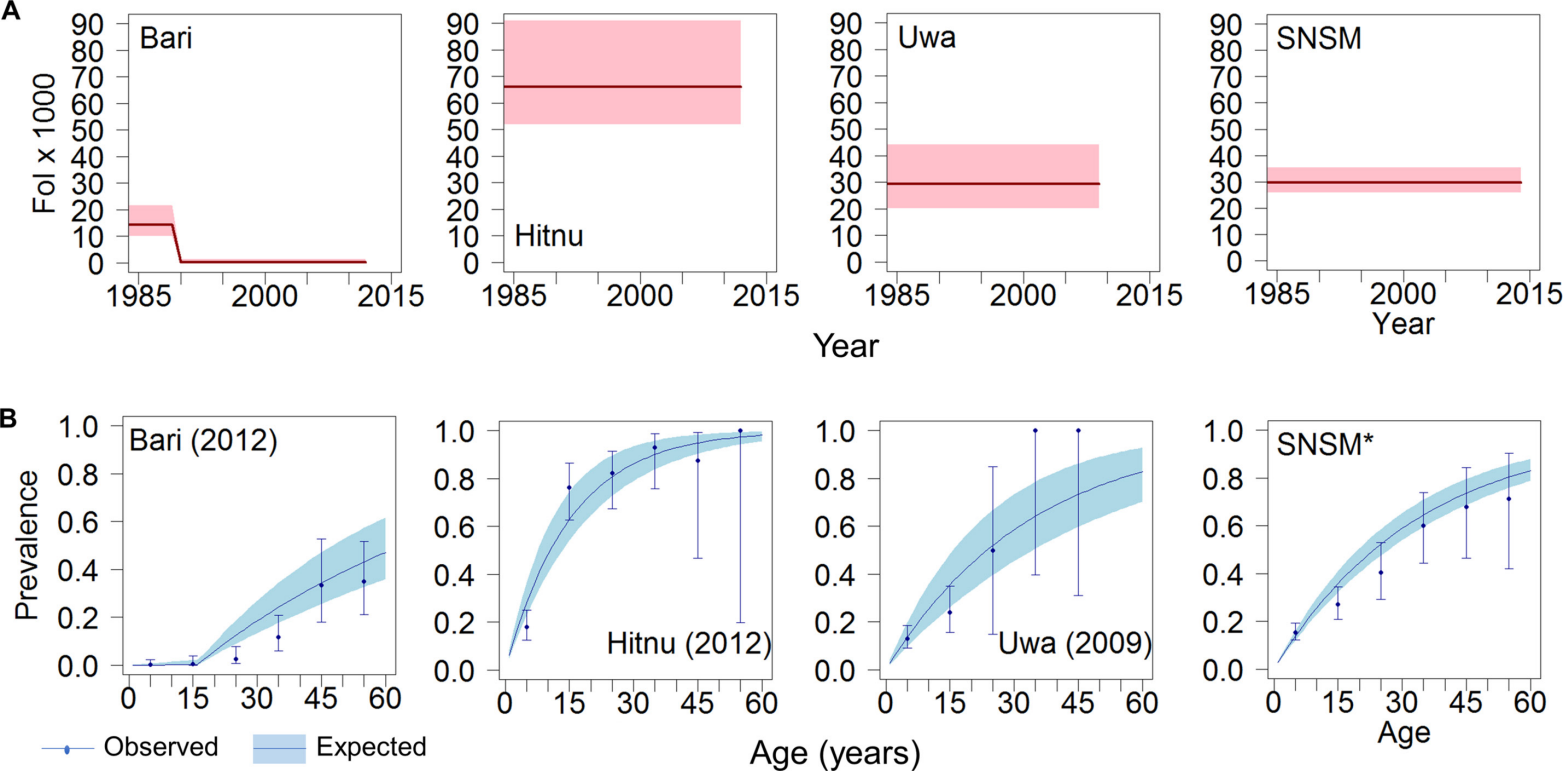
Force-of-infection (FoI) and prevalence trends of Chagas disease in four different indigenous settings in Colombia. (A) FoI per 1000 population at-risk per year vs year for the period 1984–2014. Red solid lines represent median FoI and pink shaded areas the 95% Bayesian Credible Intervals (95% BCI) according to best-fit models. (B) Age profiles of Chagas disease seroprevalence in specific years. Blue solid lines represent median predicted prevalence and coloured shaded areas the 95% BCI; solid circles represent observed prevalence values (plotted for the midpoint of each 10 year age range) and error bars are exact 95% CI. Panels from left to right correspond to four different indigenous groups, namely, Bari (Santander Norte) in 2012, Hitnu (Arauca) in 2012, Uwa (Boyacá) in 2009 and Sierra Nevada de Santa Marta (SNSM*, two serosurveys in 2007–2014).

The highest FoI values from individual serosurveys were identified for indigenous communities (figures 2 and 3). In the Hitnú communities, the FoI was 66 (95% BCI 52 to 91) based on a 2012 survey. In the SNSM communities, the FoI was 30 (95% BCI 26 to 35), based on three different surveys (2000, 2007 and 2014). In the Uwa community, the FoI was also 30 (95% BCI 20 to 44), based on a 2009 survey. For the Bari community in Santander Norte, a single intervention model (model 2) fitted the data best, suggesting that the FoI decreased from 14 (95% BCI 10 to 22) before the late 1980s to 0.1 (95% BCI 0.005 to 1.4) afterwards. The average predicted prevalence by age 15 years reached 60% in Hitnú, 36% in SNSM, 35% in Uwa and only 0.2% in Bari communities (figure 3).

### Subnational-level trends in FoI

**Figure 4:**
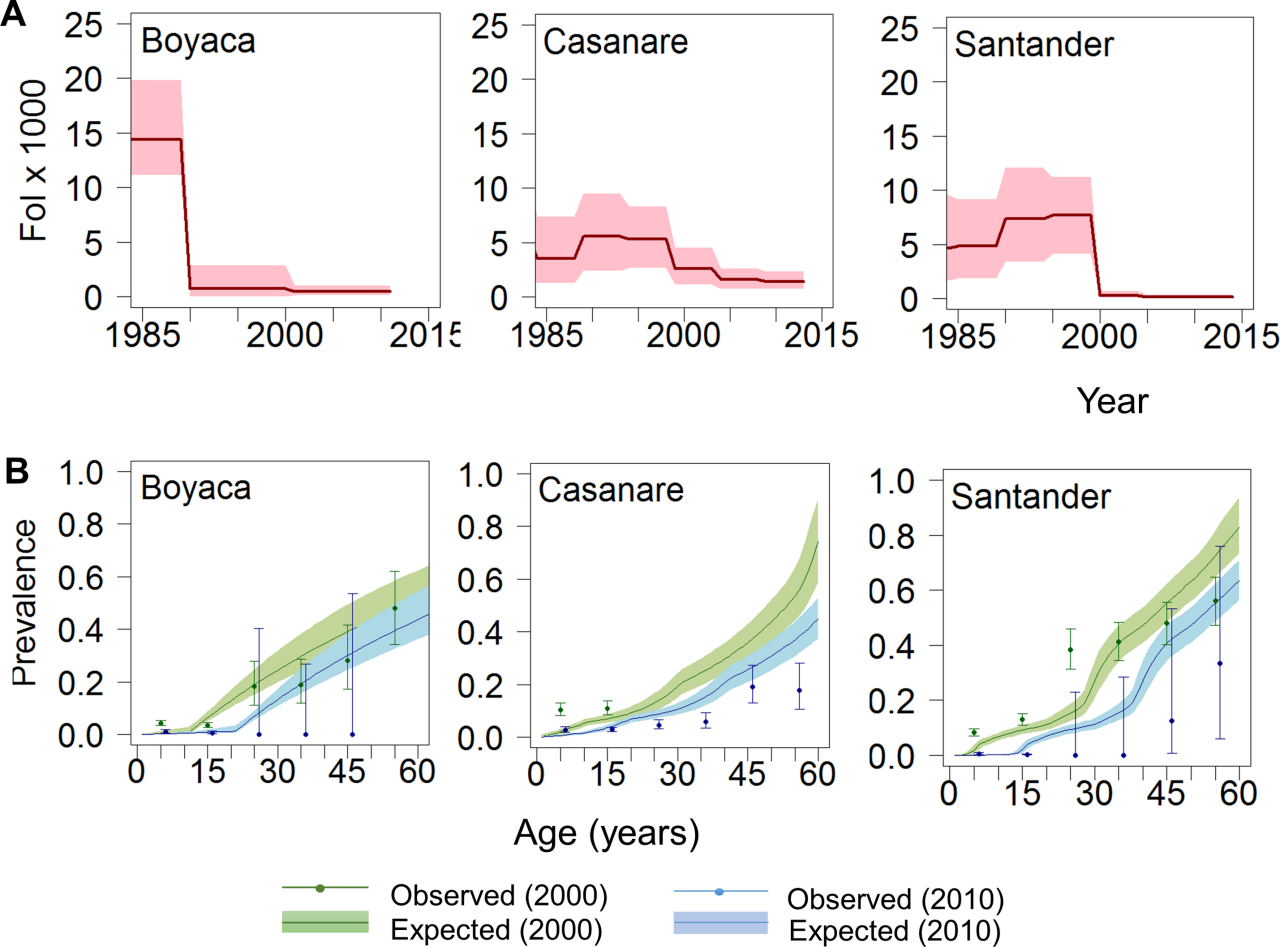
Temporal trends in the force-of-infection (FoI) and age-prevalence profiles of Chagas disease in Colombia at subnational level in three of the most endemic departments. (A) FoI per 1000 population at-risk per year vs year for the period 1984–2014. Red solid lines represent median FoI and pink shaded areas the 95% Bayesian Credible Intervals (95% BCI) according to best-fit models. (B) Age profiles of Chagas disease seroprevalence for 2000 (green) and 2010 (blue). Coloured solid lines represent median predicted prevalence and coloured shaded areas the 95% BCI; solid circles represent observed prevalence values (plotted for the midpoint of each 5-year age range) and error bars are exact 95% CI. Panels from left to right correspond to Boyacá (model 2), Casanare (model 3) and Santander (model 3).

**Figure 5:**
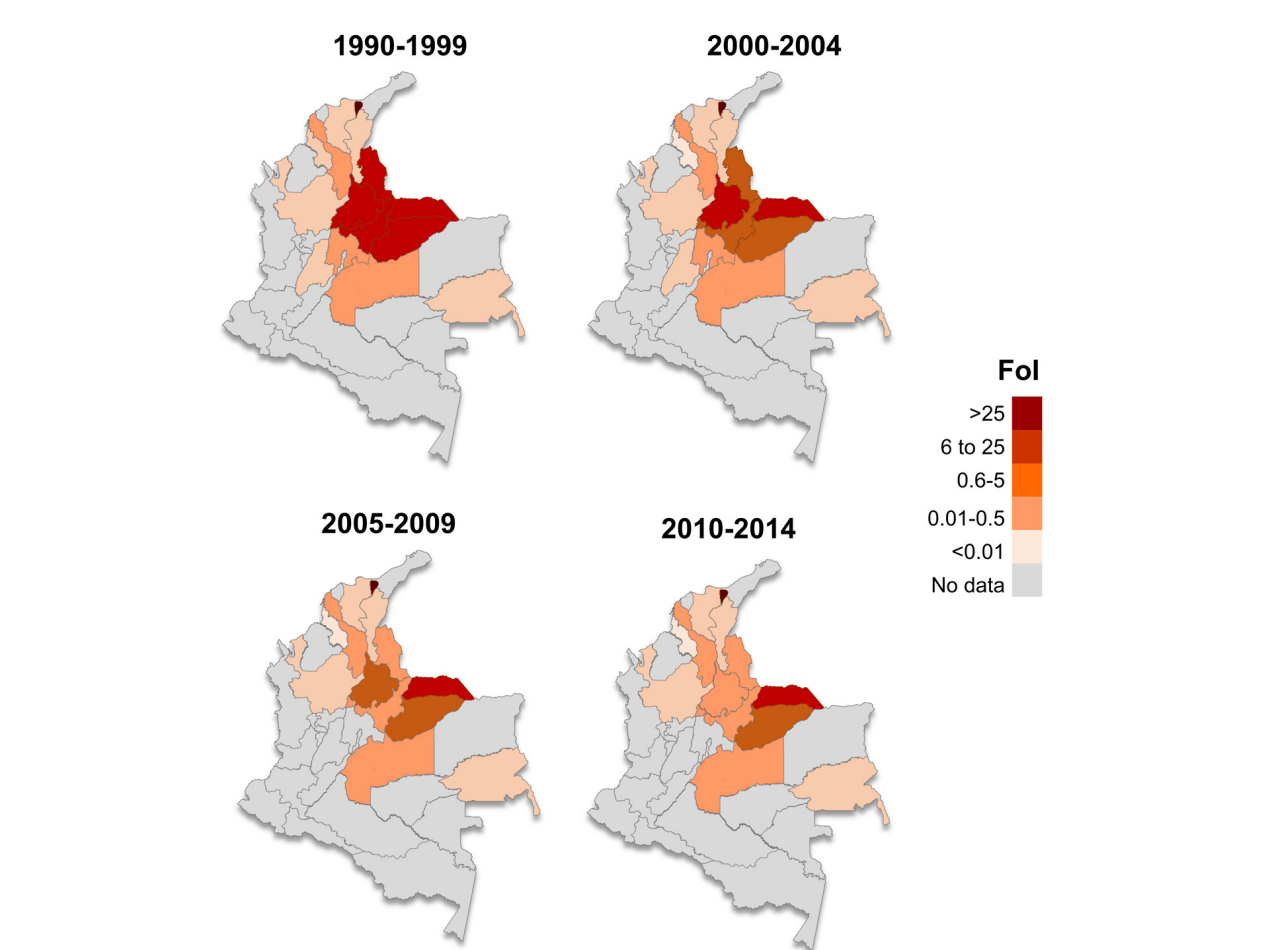
Maps illustrating spatiotemporal changes in force-of-infection (FoI) of Chagas disease in Colombia (1990–2014). Colour intensity represents the magnitude of the FoI (per 1000 population at-risk per year); for departments in grey there are no available seroprevalence data. The identification of the departments has been provided in figure 1.

Online supplementary Table 3 presents the best-fit model for each administrative department. Data and fits for Boyacá, Casanare and Santander, the departments providing most of the available information, are shown in figure 4. Boyacá and Santander have had significant reductions in FoI over time. For these departments, the FoI was currently estimated at <1 per 1000 population at-risk per year (figure 4A) and the predicted prevalence dropped sharply to <1% in children aged ≤15 years (figure 4B; expected prevalence for 2010). In Casanare, the FoI was historically somewhat lower than in Boyacá and Santander. Although a decreasing trend was also observed in this department, Casanare currently has one of the highest FoI values at departmental level, reaching 3 in 2013 (95% BCI 2 to 4), that is, more than three times greater than those of Boyacá and Santander. In contrast, for departments such as Bolivar and Guainía, the FoI remained constant over time at a low level (<1), and the predicted age prevalence profiles differ markedly from those in traditionally endemic departments (see online supplementary figure 2). Trends in the FoI at subnational level between 1990 and 2014 are presented in figure 5. Note that for the departments in grey there were no seroprevalence data available.

### Setting-level trends in FoI

When the information was analysed separately by indigenous, rural (non-indigenous) and urban settings, marked differences were observed in FoI trends, reflecting distinct epidemiological conditions.

#### Rural indigenous settings

**Figure 6:**
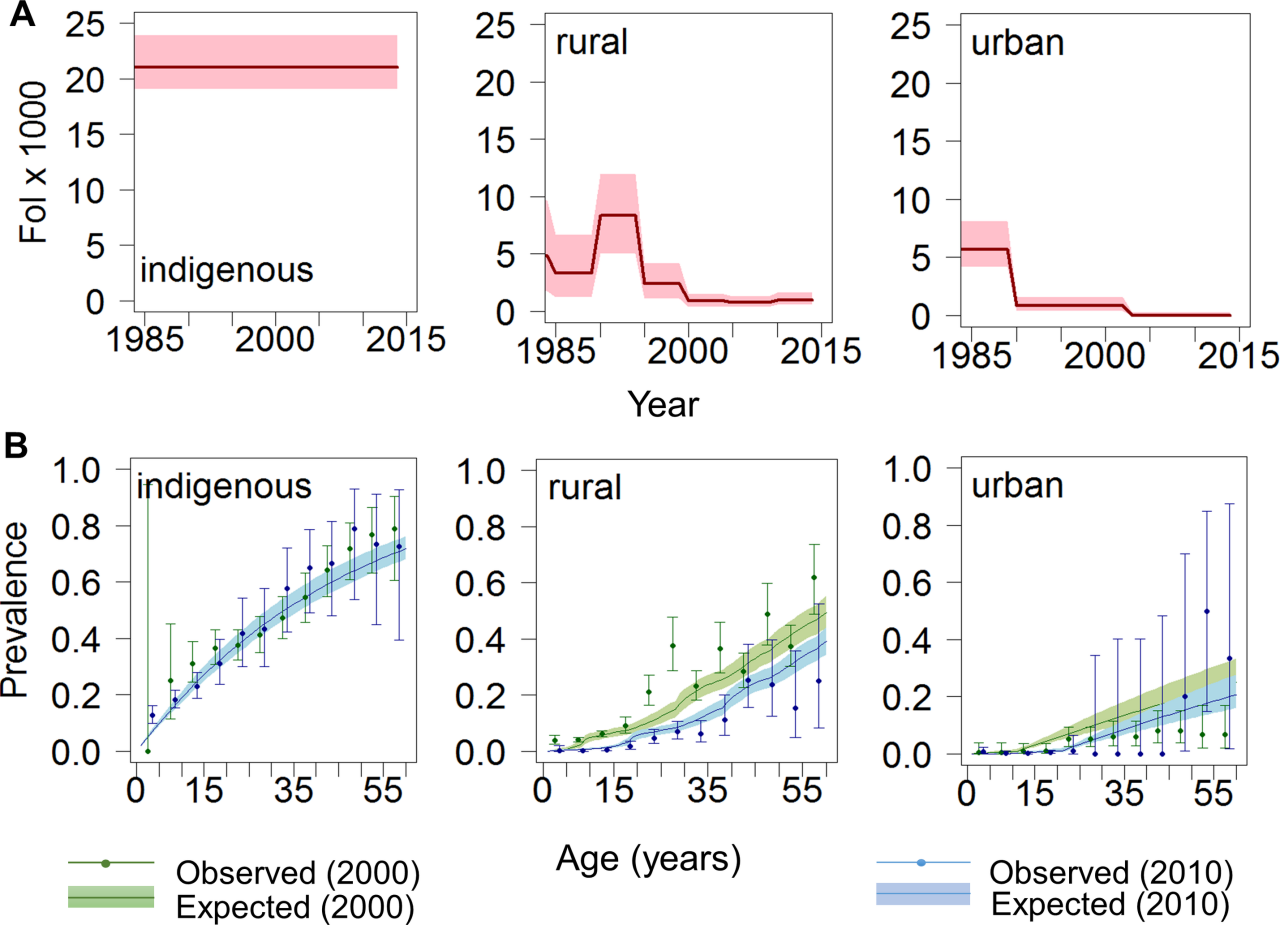
Temporal trends in force-of-infection (FoI) and age-prevalence profiles of Chagas disease in Colombia at setting level. (A) FoI per 1000 population at-risk per year vs year for the period 1984–2014. Red solid lines represent median FoI and pink shaded areas the 95% Bayesian Credible Intervals (95% BCI) according to best-fit models. (B) Age profiles of Chagas disease seroprevalence for 2000 (green) and 2010 (blue). Coloured solid lines represent median predicted prevalence and correspondingly coloured shaded areas the 95% BCI; solid circles represent observed prevalence values (plotted for the midpoint of each 5-year age range) and error bars are exact 95% CI. Left, centre and right panels correspond, respectively, to indigenous (model 1), rural non-indigenous (model 3) and urban (model 2) settings.

For the four indigenous groups, a constant FoI fitted best (model 1, online supplementary table 4), with an average FoI of 20 (95% BCI 18 to 22) (figure 6A, upper left panel). Age-prevalence profiles for the years 2000 and 2010 are shown in figure 6B, lower left panel).

#### Rural (non-indigenous) settings

A model with yearly changes in FoI (model 3) was preferred in rural communities, based on 46 serosurveys from 15 Chagas-endemic departments (see online supplementary table 4). In the early 1990s, the FoI reached up to 10 (95% BCI 8.3 to 11.9), decreasing thereafter. From 1999 onwards, the median FoI was estimated at 0.9 (0.1–2.2) (figure 6A, upper middle panel). In 2010, the predicted prevalence in those aged ≤5 years was <3%, reaching 20% (95% BCI 18% to 23%) among those aged 25 years and increasing to 44% (40%–50%) among those aged 60 years (figure 6B, lower middle panel).

#### Urban settings

Two changes in the FoI were inferred in 1995 and 2002 (model 2, online supplementary table 4) from a total of 33 serosurveys in Arauca, Boyacá, Casanare, Santander Norte and Santander. The FoI was 5.6 (95% BCI 4.2 to 8.1) before 1990, 0.8 (95% BCI 0.4 to 1.6) until 2002 and 0.02 (95% BCI 0.0004 to 0.2) from 2003 onwards (figure 6A, upper right panel). Predicted seroprevalences were lower than those in rural areas, yet >5% of adults aged 30 years were predicted to be seropositive, with prevalence reaching 19% (95% BCI 15% to 25%) among those aged 60 years (figure 6B, lower right panel).

## Discussion

Although progress has been made in Latin America towards Chagas disease control, within-country heterogeneities in transmission intensity, incidence and prevalence remain poorly recognised and quantified. Here, we used detailed retrospective analyses of seroprevalence data to quantify such temporal and spatial heterogeneities in Colombia. The estimated FoI of *Trypanosoma cruzi* (as measured by the force-of-seroconversion) in several endemic rural and urban areas of Colombia showed a reduction over the last three decades (eg, by nearly 90% in some areas of Boyacá and Santander), likely due to reductions in intradomiciliary transmission. However, high FoI persists in some areas, particularly in remote rural and indigenous settings (where the FoI can be up to 80 times greater than in non-indigenous populations of the same administrative units, eg, compare serosurveys 75–77 in online supplementary figure 2). Importantly, our results also reveal a large remnant of prevalent cases (and hence burden of disease) in adult populations throughout Colombian endemic regions due to high exposure earlier in life.

The fitted models provided reasonable predictions of seroprevalence age profiles across the surveys examined. The outputs for Boyacá and Santander are consistent with the recent recognition of local interruption of intradomiciliary transmission in some municipalities of these departments, particularly those previously infested by intradomiciliary *R. prolixus.* 19 However, such reductions in transmission were not experienced in all departments, for example, the estimated FoI for Casanare was at least three times greater than those of rural areas of Boyacá and Santander. The presence of active sylvatic transmission by *R. prolixus*, whose distribution is expanding due to landscape transformation in the region, is likely to play a role as this species is less well tackled by more traditional vector control strategies. Numerous studies have illustrated the difficulties in controlling sylvatic triatomine populations, including (1) lower effectiveness of insecticide spraying strategies and higher risk of triatomine recolonisation of dwellings,^12 18 20^ and (2) occurrence of oral transmission outbreaks.^21^ In line with this observation, the low and atypical (weakly age-related) seroprevalence profiles observed in some endemic areas of Antioquia and Bolivar—where no clear history of domiciliary transmission exists^22^ —may also be due to sylvatic and oral transmission by secondary vectors such as *Rhodnius pallescens* or *Panstrongylus geniculatus.* 23–27 Finally, the observed incidence trends in urban settings are likely to reflect migration from rural areas to towns and cities, as such settings are deemed unlikely to sustain active vectorial transmission. Although the magnitude of rural to urban migration has been well documented (from 70% of the Colombian population living in rural areas during the 1940s to 30% currently),^28^ the role of population movement in shaping the epidemiology of Chagas disease in endemic countries is still poorly understood.^29 30^

Serology, as a diagnostic technique for Chagas disease, has a number of limitations (particularly for detecting the acute phase), including problems of interpretation as a direct marker of infection, unknown relationship with disease progression and cure and not well-understood rates of seroreversion.^6 31^ Although the diagnosis of *T. cruzi* infection has improved in recent years, especially with the development of PCR-based molecular techniques, their limited sensitivity in the chronic phase (57%–64%) and high cost have confined their use to specialised laboratories and prevented their field testing to replace traditional serology.^32^ Therefore, and despite its limitations, seroprevalence surveys remain relevant, affordable and relatively easy to implement in endemic settings, permitting the assessment of transmission patterns and trends, as demonstrated in other studies.^11 32–34^ However, since different serodiagnostic assays differ in their sensitivity and specificity, it will be crucial to adjust estimates according to the diagnostic performance of the tests used in the various serosurveys (as implemented in this study).

In Peru and Bolivia, catalytic models based on serological surveys conducted at small scales (eg, within a city), allowed identification of specific years when interruption of *T. cruzi* transmission was achieved,^33^ assessment of the impact of reinvasion by triatomines once vector control was stopped^34^ and characterisation of local introduction and spread at fine spatial scales, helping to identify microepidemics.^35^ Using aggregate data at a national scale, Feliciangeli *et al* showed the potential of FoI models to understand Chagas disease trends in Venezuela from 1940 to 2000.^12^

While an aggregate approach at a national level has the advantage of providing a broader picture, it has the disadvantage of potentially masking important underlying heterogeneities that need to be highlighted. We attempted to bridge the gap between microepidemiological and macroepidemiological scales by analysing 109 serosurveys individually and subsequently aggregating them at subnational and setting scales. In doing so, we acknowledge a risk of observing survey-specific heterogeneities rather than true underlying temporal and/or spatial patterns on the one hand, and on the other assuming homogeneity within administrative units or settings. We did not, however, aggregate the data at a national level, as this could lead to erroneous conclusions regarding the impact of Chagas disease control in Colombia. Besides, there are still areas of the country where seroprevalence studies have not been conducted or are not yet available. Ideally, surveys should be conducted not by simple random sampling, but by adaptive sampling methods^36^ in endemic areas, including those where information is still lacking.

Previous studies have informed that the main vector of Chagas disease in Colombia, *R. prolixus,* has restricted its geographical distribution in recent years, possibly due to control actions (insecticide spraying, education programmes) mainly carried out by the secretaries of health of the departments of Boyacá, Cundinamarca and Santander; along with a reported expansion of the same species’ distribution across the oriental plains of the country (departments of Casanare and Meta particularly), where palms cultures have been extended and sylvatic transmission takes place.^37^ Interestingly, as *R. prolixus* is mostly a so-called introduced species across the Andean region in Colombia, this has a much greater potential to be eliminated (and interruption transmission achieved after basic control strategies) that in comparison with the oriental plains region or even Venezuela, where *R. prolixus* is mostly considered an autochthonous species.^38^ Unfortunately, detailed data about specific control interventions undertaken in the different locations across the three decades investigated in this paper are scarce, non-centralised and therefore not available in public repositories nor through the Ministry of Health. On the other hand, major changes in terms of housing living conditions in some rural areas of the country have also been resulted from housing improvement programmes in the last three decades.^28^ Indeed, two recent seroprevalence studies in different locations have shown that adults in Chagas endemic areas used to live in poorer housing conditions two decades ago (ie, 40%–47% exposed to soil floor in childhood vs 9%–12% in present days^39 40^). We hypothesise that these structural changes could also potentially play a role towards the reduction of domiciliary transmission in some locations identified in this study. Such information on vector control surveillance and socioeconomic conditions would certainly help confirm and explain the findings of this research and further investigate the underlying causes of historical changes in the FoI and to assess the real potential for sustained elimination.

## Conclusion

FoI models such as those applied here could be used to analyse retrospectively many of the serosurveys already available in Latin America to quantify incidence trends and obtain age-specific prevalence profiles that would allow assessment of whether serological (or other) criteria for intradomiciliary transmission interruption have been met. Such a relatively low-cost exercise (compared with the collection of longitudinal data), could help endemic countries to quantify the impact of (historical and/or current) interventions, estimate the number of prevalent cases and ensuing disease burden, conduct cost-effectiveness and economic evaluations^41^ and ultimately assess whether they are on track to reach the WHO 2020 goals. Despite the evident progress in some regions, given the marked heterogeneity in the FoI found in this research, between rural and urban areas and between indigenous and non-indigenous settings, it is unlikely that the goal of achieving interruption of domiciliary transmission in all endemic areas of Colombia can be met by 2020. Greater efforts should be made in terms of improving vector surveillance, detailed records of interventions undertaken and conducting seroprevalence studies, especially in areas where there is not recent information available. Also, given the results of this research, for future serosurveys in Latin America, it would be advisable to include socioeconomic variables in adaptive sampling designs, and ideally should be conducted with representativeness at subnational level, especially when transmission interruption is under evaluation. This would greatly assist endemic countries’ efforts towards Chagas disease prevention, control and care in the region.
