# Supplementary material for: Modelling historical changes in the force-of-infection of Chagas disease to inform control and elimination programmes: application in Colombia
Source: BMJ Glob Health. 2017 Sep 7;2(3):e000345. doi: 10.1136/bmjgh-2017-000345 (PMC5680445; doi:10.1136/bmjgh-2017-000345)
Supplement: Supplementary file 1 [file bmjgh-2017-000345supp001.pdf]

Supplementary Data for

Modelling historical changes in the Force-of-Infection of Chagas disease to inform control and elimination programmes: application in Colombia

Zulma M. Cucunubá*, Pierre Nouvellet, Lesong Conteh, Mauricio Javier Vera, Víctor Manuel Angulo, Juan Carlos Dib, Gabriel Parra-Henao, and María-Gloria Basáñez

***Corresponding author**
Zulma M. Cucunubá
Department of Infectious Disease
Epidemiology & London Centre for Neglected
Tropical Disease Research
Faculty of Medicine (St Mary's campus)
Imperial College London, Norfolk Place
London W2 1PG
Tel: +44 (0) 207 5943229
Fax: +44 (0) 207 4023927
zulma.cucunuba@imperial.ac.uk

This file includes:

Supplementary Methods

Text A. Systematic Review of Serological Surveys.

Text B. Likelihood Expression.

Text C. Adjustment of Seroprevalence by Diagnostic Tests.

Text D. Exploring Spatial Correlation.

Supplementary Figures

Supplementary Figure 1. Flowchart of the systematic review conducted to identify and select the serosurveys included in this study.

Supplementary Figure 2. FoI per 1,000 population at risk per year vs. year for the period 1984–2014 for 109 individual serosurveys.

Supplementary Figure 3. Semivariogram analysis of spatial trend in the FoI for the years a) 1995, b) 2000, c) 2005 and d) 2010.

Supplementary Tables

Supplementary Table 1. Characteristics of the serosurveys included in the analyses.

Supplementary Table 2. Deviance Information Criterion for selecting the best model fit for the force-of-infection of Chagas disease in Colombia in individual serosurveys.

Supplementary Table 3 Deviance Information Criterion for selecting the best model fit for subnational-level trends in the force-of-infection of Chagas disease in Colombia.

Supplementary Table 4. Deviance Information Criterion for selecting the best model fit for setting-level trends in the force-of-infection of Chagas disease in Colombia.

Supplementary Table 5. Comparison of Deviance Information Criterion for selecting the best model fit for aggregated vs. individual serosurveys in Colombia at subnational level and in four different settings

Supplementary References

Supplementary Methods

Text A. Systematic Review of Serological Surveys

We conducted a systematic review following the PRISMA guidelines. We systematically searched PubMed, Web of Knowledge datasets, and the Latin American Index (LILACS—Literatura Latinoamericana de Información en Ciencias de la Salud, in Spanish) datasets up to 30 September 2015, with no restrictions applied for year and language of publication using the following terms algorithm ("seroepidemiologic studies"[MeSH Terms] OR "cross-sectional"[All Fields] OR "estimates"[All Fields] OR "seroprevalence"[All Fields] OR "serosurvey"[All Fields] OR "survey"[All Fields] OR "prevalence"[MeSH Terms]) AND ("chagas disease"[MeSH Terms] OR "trypanosoma cruzi"[MeSH Terms]) AND ("colombia"[MeSH Terms] OR "colombia"[All Fields]).

For LILACS database we used the following terms: tw:((chagas disease OR enfermedad de chagas OR doença de chagas) AND colombia AND (prevalence OR seroprevalence OR prevalencia OR prevalência)) AND (instance:"regional").

A flowchart of literature selection process is depicted in Supplementary Figure 1. The details of the literature used in the analyses are listed in Supplementary Table 1.

Text B. Likelihood Expression

The catalytic models used in the analysis are described in the main text. Here we provide further explanation on the likelihood expression.

Using seroprevalence (binomial) data, and assuming homogeneity across spatial units, for a total of S surveys within each unit and each component survey s encompassing N_s age classes, the likelihood (L) of the parameter(s) of interest (θ) given data on the number of seropositives c, the total number tested n, and their age a, is,

$$L(\theta | c,n,a) \propto \prod_{s=1}^{S} \left[\prod_{a=1}^{N_s} p_{a,\tau,s}{}^{c_{a,s}} \left(1 - p_{a,\tau,s}\right)^{n_{a,s} - c_{a,s}} \right], \quad \text{(S1)}$$

where $c_{a,s}$ and $(n_{a,s} - c_{a,s})$ are, respectively, the number of seropositive and seronegative individuals in age class a for survey s, $p_{a,\tau,s}$ corresponds to equations (1–3) in the main text (after adjustment for diagnostic performance as explained in Text C), and θ is the set of parameter(s) to be estimated (λ in Model 1, up to five values of λ and up to four values of ξ in Model 2, and $\{\lambda_i\}$ in Model 3).

Text C. Adjustment of Seroprevalence by Diagnostic Tests

Given that the results of the serosurveys depend on the intrinsic diagnostic performance characteristics (*Sensitivity* and *Specificity*) of the assays used, these properties [1] were used to adjust the expected (modelled) seroprevalence using the following expression,

$$p = sens \times P + (1 - spec) \times (1 - P) \,, \qquad \text{(S2)}$$

where p is the 'apparent' prevalence, P is the modelled 'true' prevalence; and $sens$ and $spec$ are, respectively, the test sensitivity and specificity [2, 3].

Sequential testing

In some cases, multiple testing was used in the seroprevalence studies. Typically, for Chagas disease screening, this process is done sequentially (second test, B, performed only for those who tested positive on the first test, A) rather than simultaneously. This sequential testing modifies the net values of sensitivity and specificity, with an overall reduction in sensitivity and an increase in specificity. Based on Bayes theorem, the calculations of net specificity and net sensitivity for sequential testing are as follows [4].

Using the multiplication rule, $P\,(A \cap B) = P(A) \text{ x } P(B \mid A)$. When the two events (in this case the diagnostic performances of the tests) are independent it follows that, $P\,(B \mid A) = P(B)$. Therefore, $P\,(A \cap B) = P(A) \text{ x } P(B)$, and $NetSensitivity = (A)_{sens} \text{ x } (B)_{sens}$.

By the addition rule of probability, $P(A \text{ U } B) = P(A) + P(B) - P\,(A \cap B)$. Therefore, $NetSpecificity = (A)_{spec} + (B)_{spec} - [(A)_{spec} \text{ x } (B)_{spec}]$. The expression for 'apparent' prevalence (p) becomes,

$$p = NetSensitivity \times P + (1 - NetSpecificity) \times (1 - P) \qquad \text{(S3)}$$

In cases where three tests were used, given that information on the type and sequence between the second and/or the third tests was not given in the original data sources, we used for the second test the values of sensitivity and specificity for IFAT

(immunofluorescence antibody test), the most commonly used second test for Chagas disease diagnosis.

Text D. Exploring Spatial Correlation

We explored spatial correlation between the values of the force-of-infection (FoI) derived from the serological surveys (using Model 3 in the main text) by means of the semivariogram method. A semivariogram is a tool used in model-based geostatistics to represent spatial correlation in observations measured at sample locations [5,6]. It is commonly presented as a graph that shows the variance versus distance between all pairs of sampled locations. The expected squared difference between two data values separated by a distance vector **h** is the variogram. The semivariogram $\gamma(\mathbf{h})$ is one half of the variogram $2\gamma(\mathbf{h})$.

Let $N(\mathbf{h})$ represent the total number of pairs of FoI values separated by lag **h** (plus or minus the lag tolerance). We can then compute the semivariogram statistics for lag **h** as,

$$\gamma(\mathbf{h}) = \frac{1}{2N(\mathbf{h})} \sum_{\alpha=1}^{N(\mathbf{h})} \left[z(\mathbf{u}_\alpha) - z(\mathbf{u}_\alpha + \mathbf{h}) \right]^2 , \qquad \text{(S4)}$$

where, $\mathbf{u}$ is the vector of spatial coordinates for α locations; $z(\mathbf{u})$ is the variable under consideration as a function of spatial location (in our case the FoI); $\mathbf{h}$ is the lag vector representing separation between two spatial locations and $z(\mathbf{u} + \mathbf{h})$ is the lagged version of the variable under consideration. The resulting semivariance plotted versus the lag is the semivariogram.

Empirical semivariograms were obtained by randomly reassigning the values of FoI at data locations for each permutation. Models were fitted to the empirical semivariogram using a number of functions (e.g. exponential, Gaussian, spherical). If the empirical semivariogram lies outside the randomly generated region, there is a significant spatial trend present in the data, and there is no evidence of spatial trend otherwise (Figure B).

For all the analysed years, the empirical variograms lie inside this region and, therefore, there is no significant spatial correlation between the surveys.

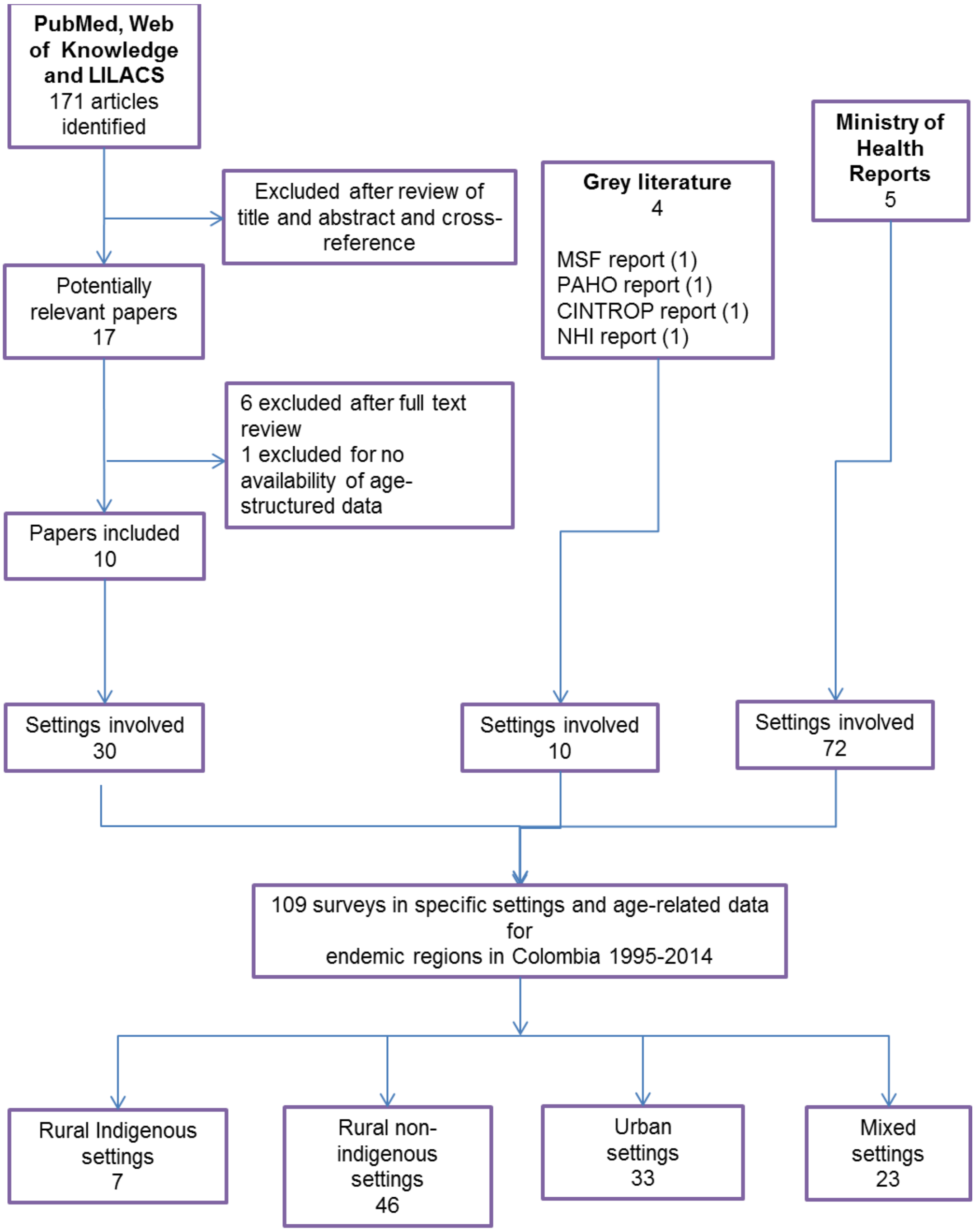

Supplementary Figure 1. Flowchart of the systematic review conducted to identify and select the serosurveys included in this study. Abbreviations: LILACS, Literatura Latinoamericana de Información en Ciencias de la Salud; MSF, Médicos Sin Fronteras; PAHO, Pan American Health Organization; CINTROP, Centro de Investigaciones en Enfermedades Tropicales; NHI, National Health Institute, Colombia.

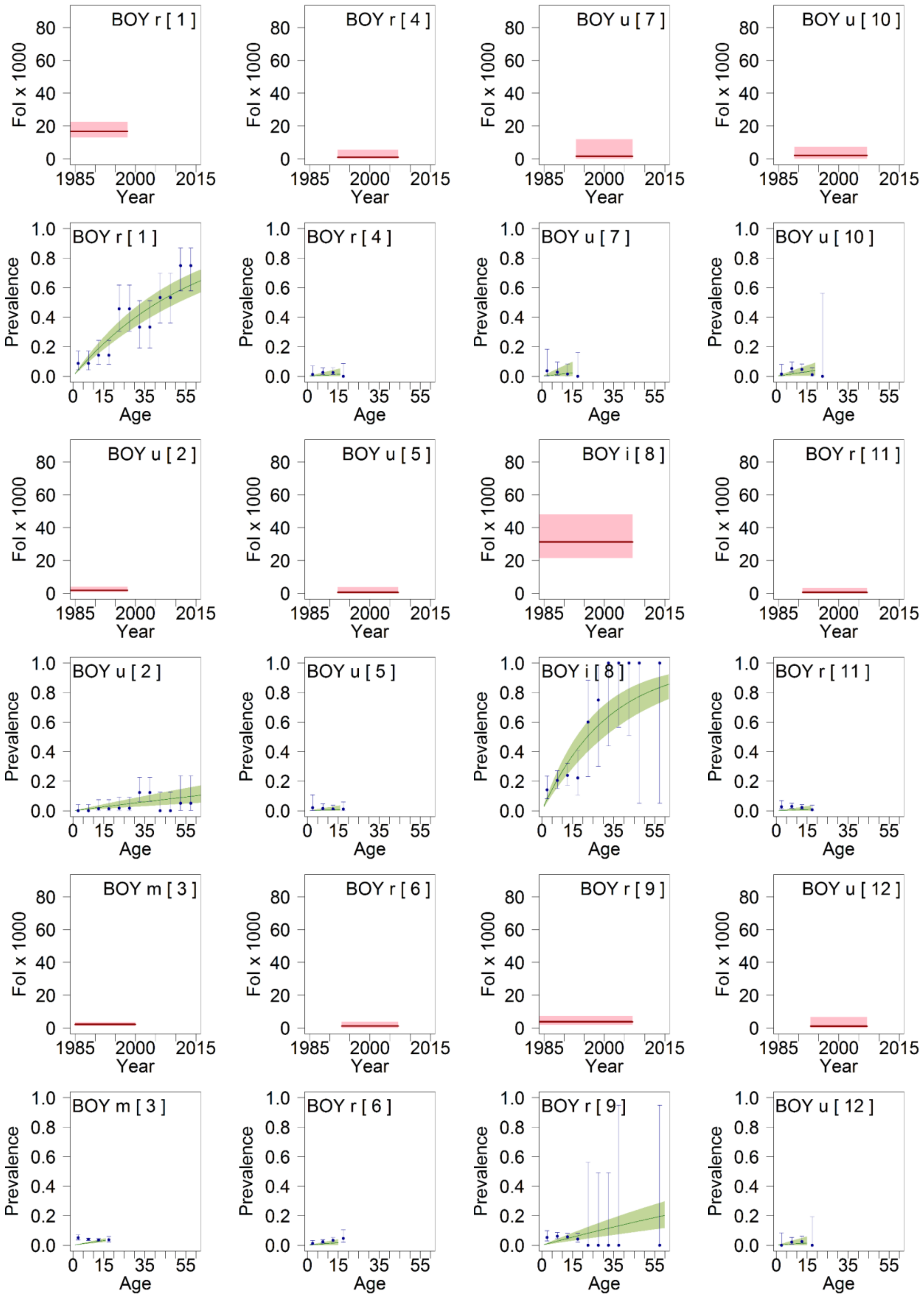
BOY r [1]
BOY r [4]
BOY u [7]
BOY u [10]
BOY u [2]
BOY u [5]
BOY i [8]
BOY r [11]
BOY m [3]
BOY r [6]
BOY r [9]
BOY u [12]
FoI x 1000
Prevalence
Year
Age

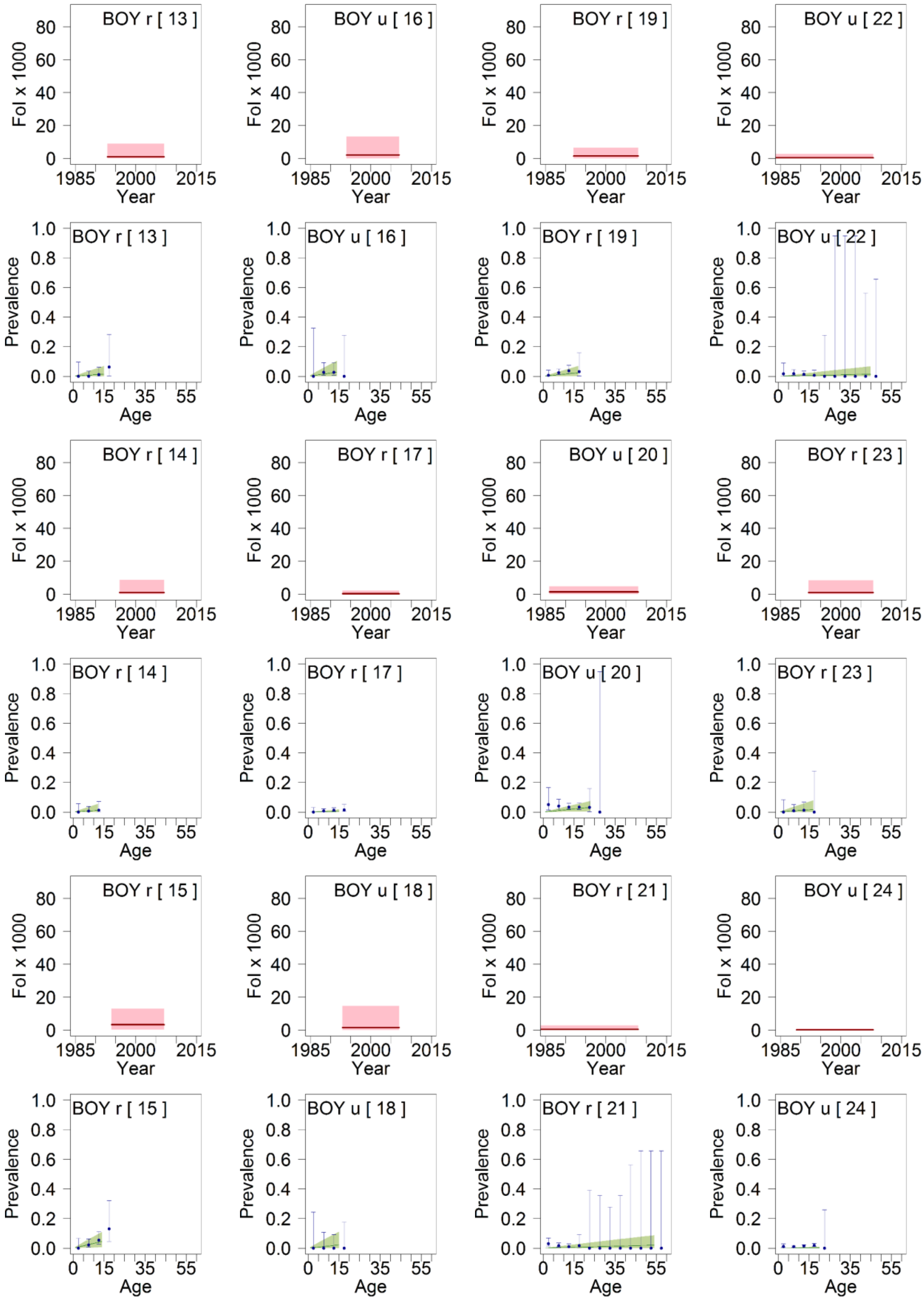

BOY r [13]
BOY u [16]
BOY r [19]
BOY u [22]
BOY r [14]
BOY r [17]
BOY u [20]
BOY r [23]
BOY r [15]
BOY u [18]
BOY r [21]
BOY u [24]
FoI x 1000
Year
1985
2000
2015
0
20
40
60
80
Prevalence
Age
0
15
35
55
0.0
0.2
0.4
0.6
0.8
1.0

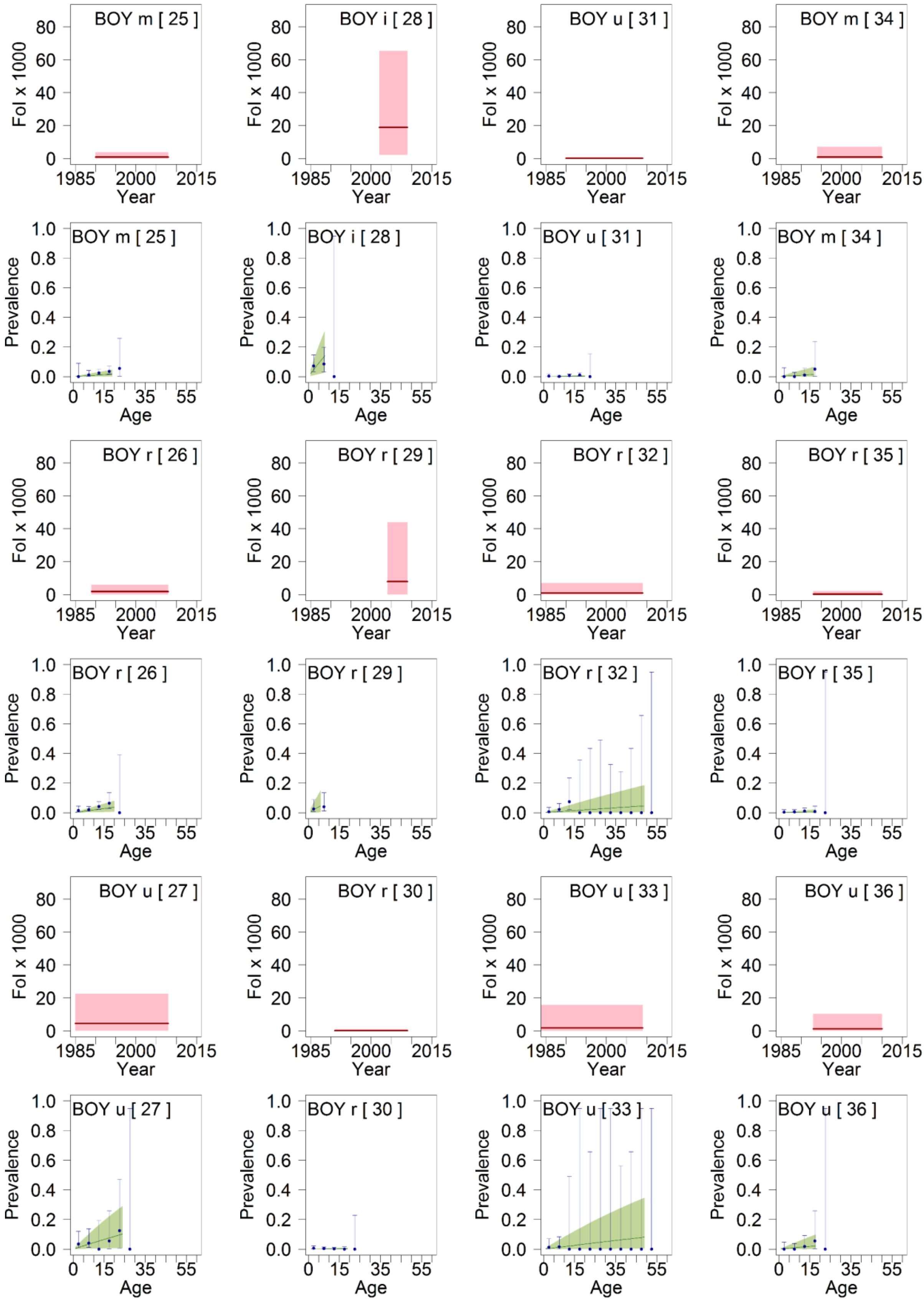

BOY m [25]
BOY i [28]
BOY u [31]
BOY m [34]
BOY r [26]
BOY r [29]
BOY r [32]
BOY r [35]
BOY u [27]
BOY r [30]
BOY u [33]
BOY u [36]
FoI x 1000
Year
1985 2000 2015
0 20 40 60 80
Prevalence
Age
0 15 35 55
0.0 0.2 0.4 0.6 0.8 1.0

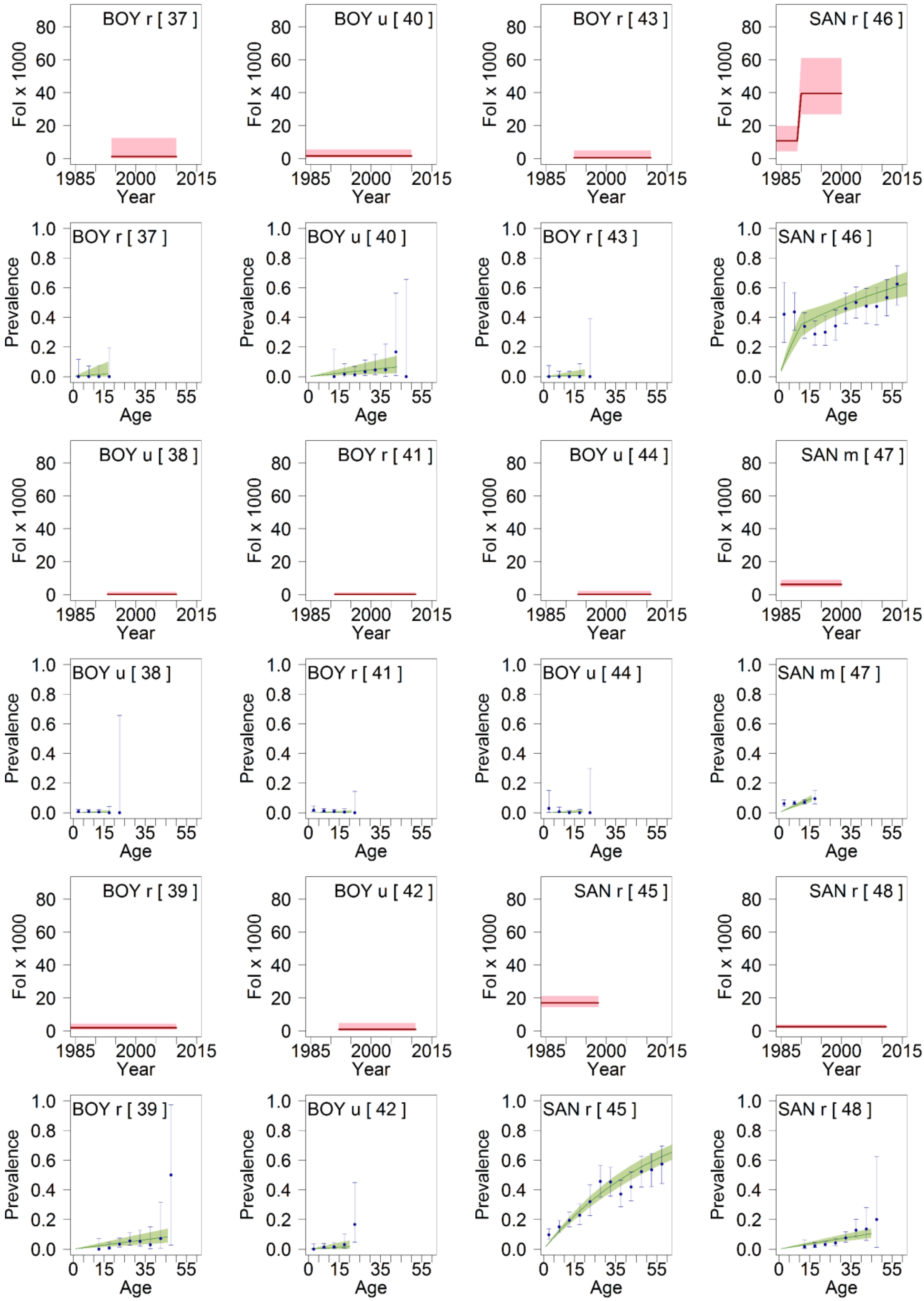
BOY r [37]
BOY u [40]
BOY r [43]
SAN r [46]
BOY u [38]
BOY r [41]
BOY u [44]
SAN m [47]
BOY r [39]
BOY u [42]
SAN r [45]
SAN r [48]
FoI x 1000
Year
Prevalence
Age
0
20
40
60
80
1985
2000
2015
0.0
0.2
0.4
0.6
0.8
1.0
15
35
55

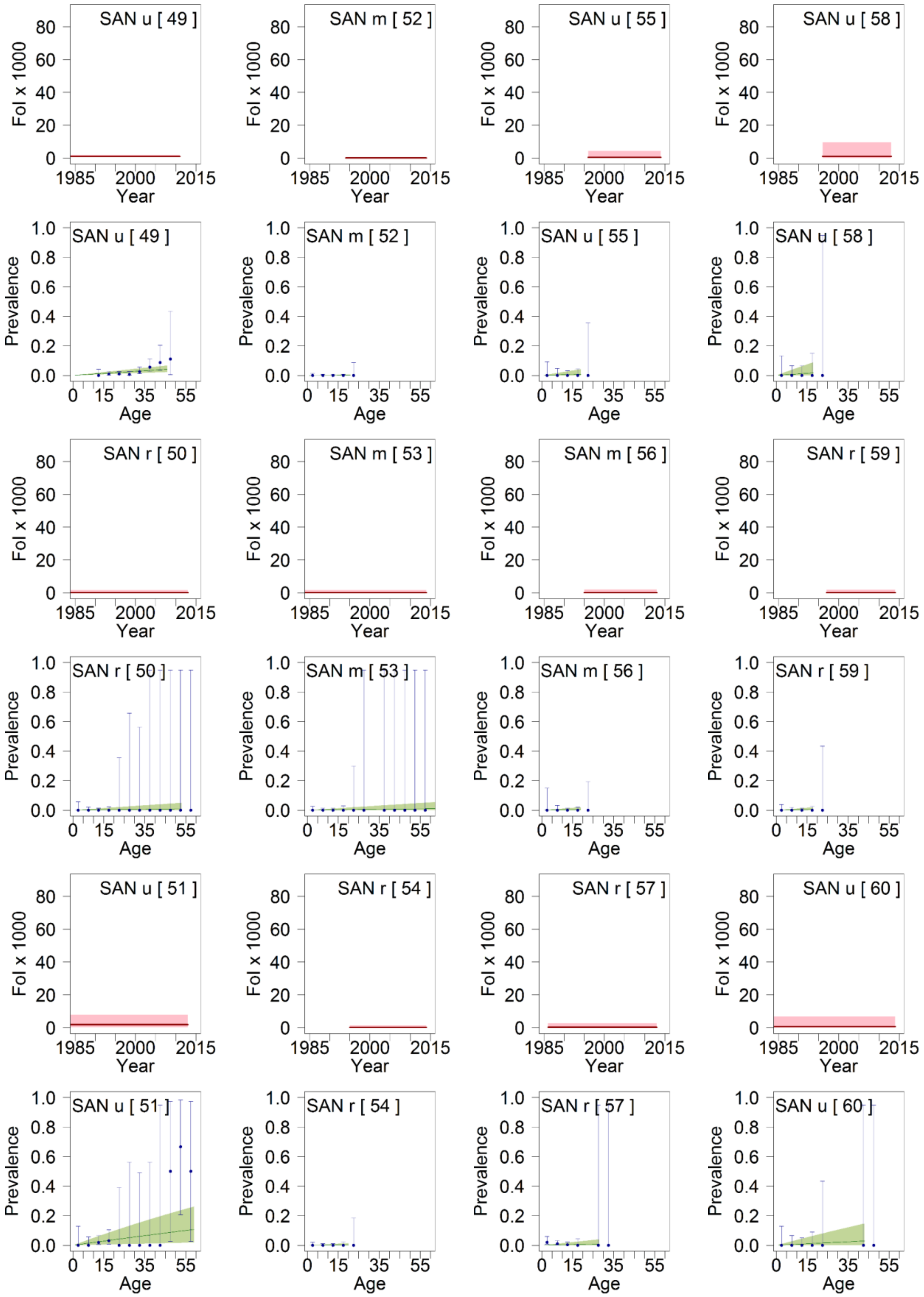
SAN u [49]
SAN m [52]
SAN u [55]
SAN u [58]
SAN r [50]
SAN m [53]
SAN m [56]
SAN r [59]
SAN u [51]
SAN r [54]
SAN r [57]
SAN u [60]
FoI x 1000
Year
1985
2000
2015
Prevalence
Age
0
15
35
55

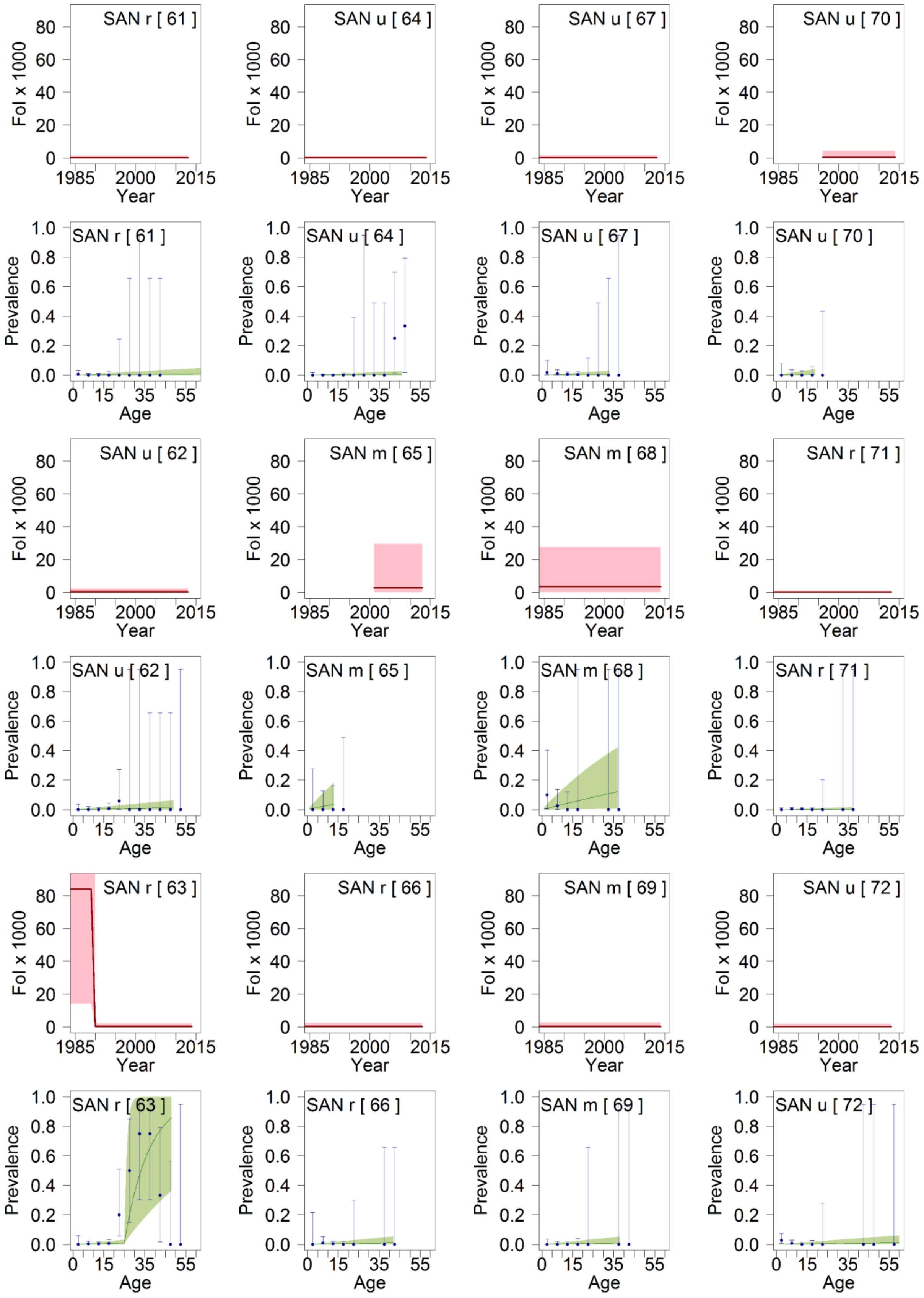

SAN r [61]
SAN u [64]
SAN u [67]
SAN u [70]
SAN u [62]
SAN m [65]
SAN m [68]
SAN r [71]
SAN r [63]
SAN r [66]
SAN m [69]
SAN u [72]
FoI x 1000
Year
Prevalence
Age

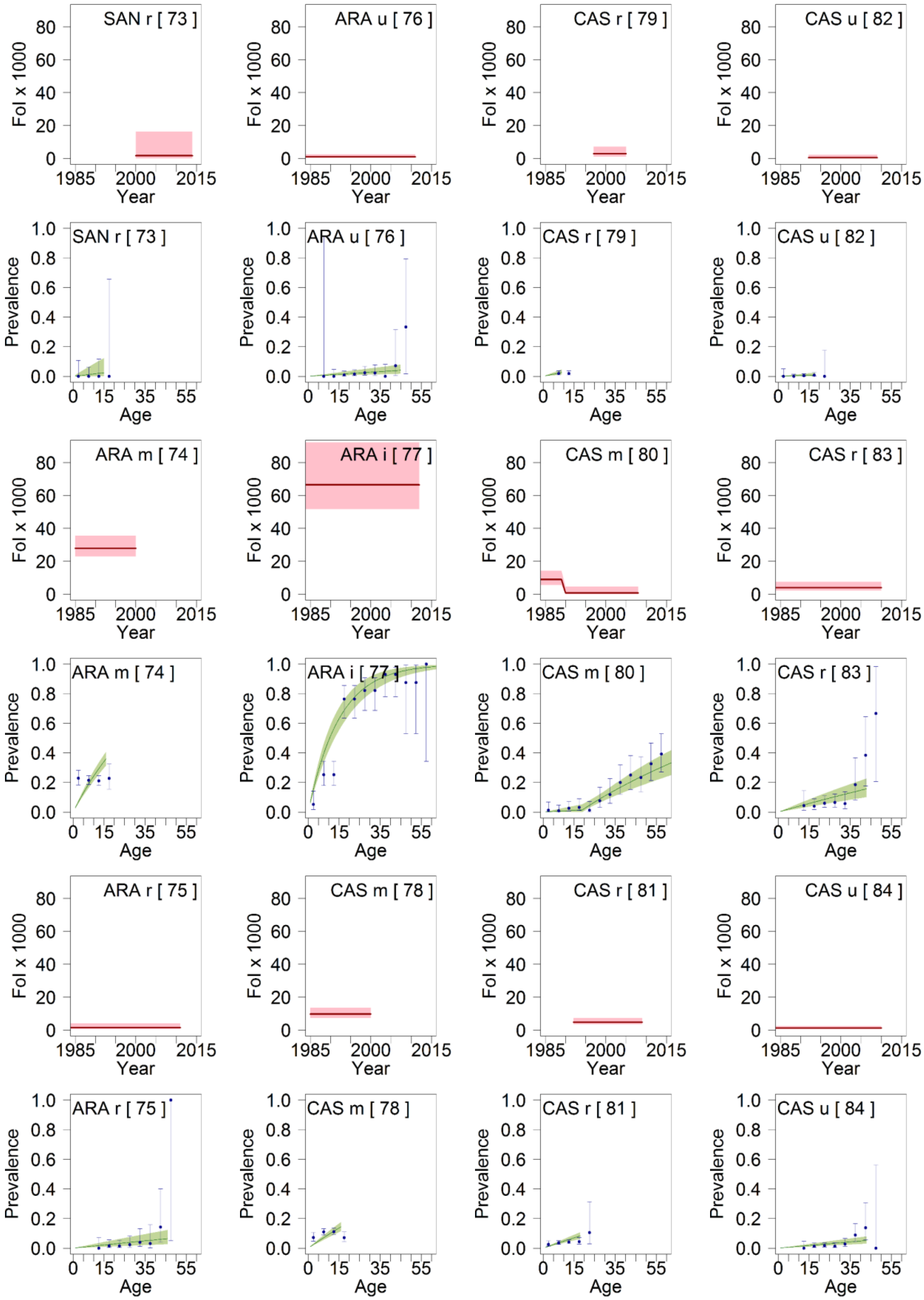
SAN r [73]
ARA u [76]
CAS r [79]
CAS u [82]
ARA m [74]
ARA i [77]
CAS m [80]
CAS r [83]
ARA r [75]
CAS m [78]
CAS r [81]
CAS u [84]
FoI x 1000
Year
1985
2000
2015
0
20
40
60
80
Prevalence
Age
0
15
35
55
0.0
0.2
0.4
0.6
0.8
1.0

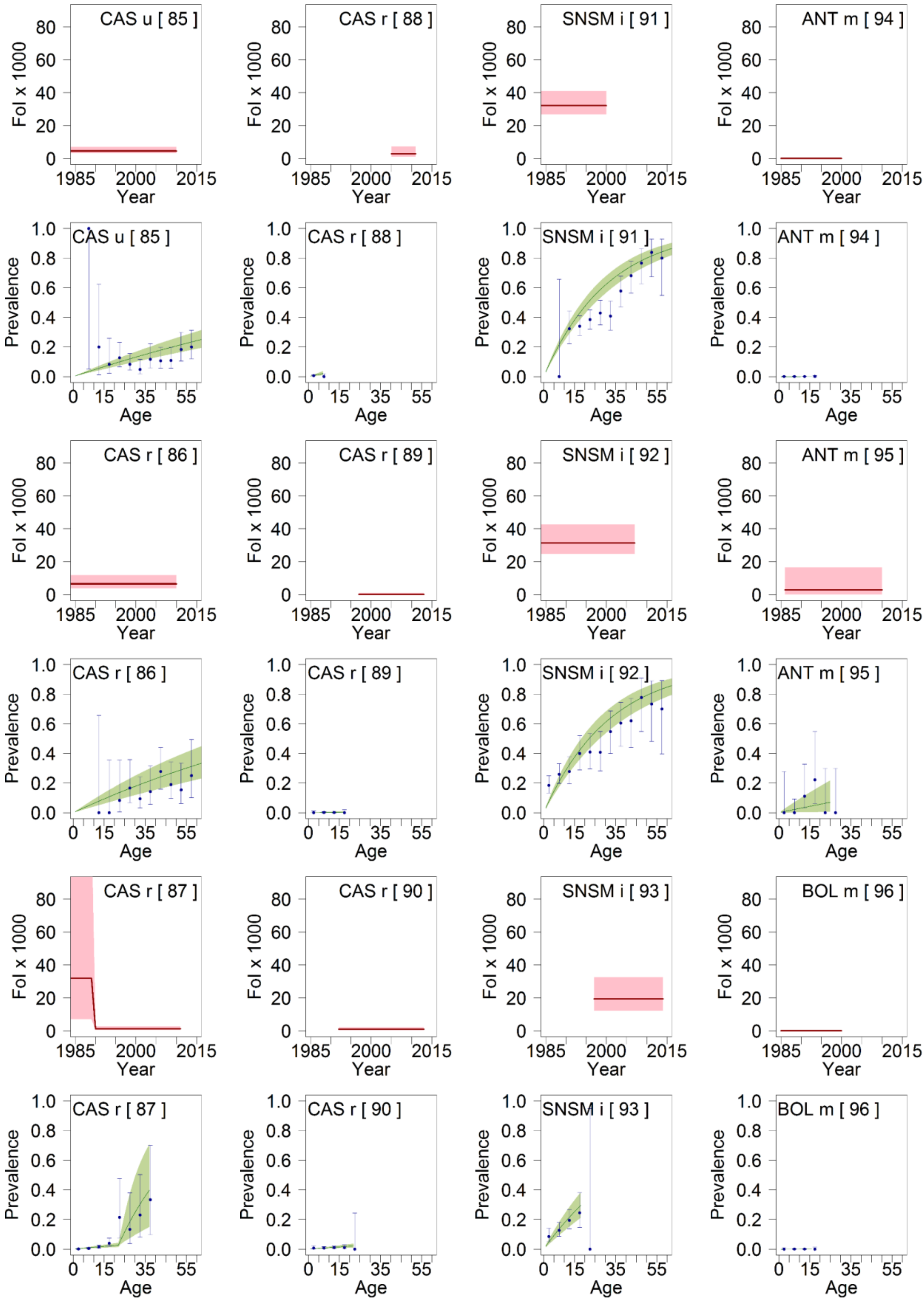

CAS u [85]
CAS r [88]
SNSM i [91]
ANT m [94]
CAS r [86]
CAS r [89]
SNSM i [92]
ANT m [95]
CAS r [87]
CAS r [90]
SNSM i [93]
BOL m [96]
FoI x 1000
Prevalence
Year
Age
1985
2000
2015
0
15
35
55
0
20
40
60
80
0.0
0.2
0.4
0.6
0.8
1.0

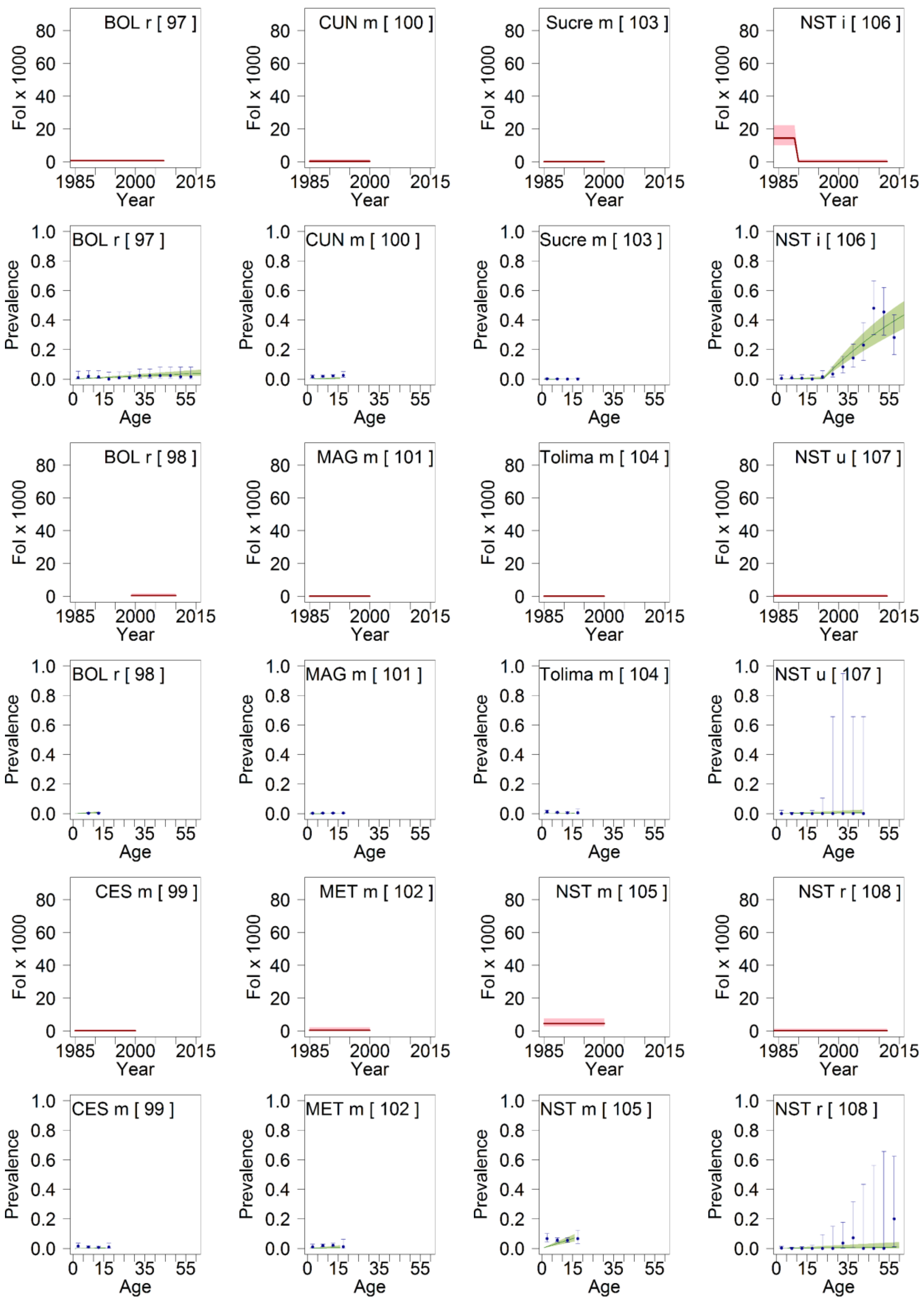

BOL r [97]
CUN m [100]
Sucre m [103]
NST i [106]
BOL r [98]
MAG m [101]
Tolima m [104]
NST u [107]
CES m [99]
MET m [102]
NST m [105]
NST r [108]
FoI x 1000
Prevalence
Year
Age

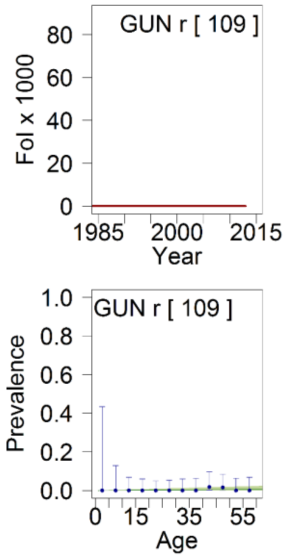

Supplementary Figure 2. Paired (upper and lower) plots for 109 individual serosurveys of the force-of-infection (FoI) per 1,000 population at risk per year vs. year for the period 1984–2014 (upper) and age profiles of Chagas disease seroprevalence (lower) for the survey year (See Supplementary Table 1). The red solid lines represent the median FoI and shaded pink areas the 95% Bayesian Credible Intervals (95% BCI) according to the best-fit models. Blue solid lines represent the median predicted prevalence and the green shaded areas the 95% BCI; solid circles represent observed prevalence values (plotted for the mid-point of each 10-year age range) and error bars are exact 95% CI. Abbreviations: BOY, Boyacá; SAN, Santander; CAS, Casanare; SNSM, Sierra Nevada de Santa Marta; ANT, Antioquia; BOL, Bolívar; CES, Cesar; CUN, Cundinamarca; MAG, Magdalena; MET, Meta; NST, Santander Norte; GUN, Guainía; i, indigeneous setting; r, rural setting; u, urban setting; m, mixed setting. Numbers in square brackets correspond to each serosurvey as cited in Supplementary Table 1.

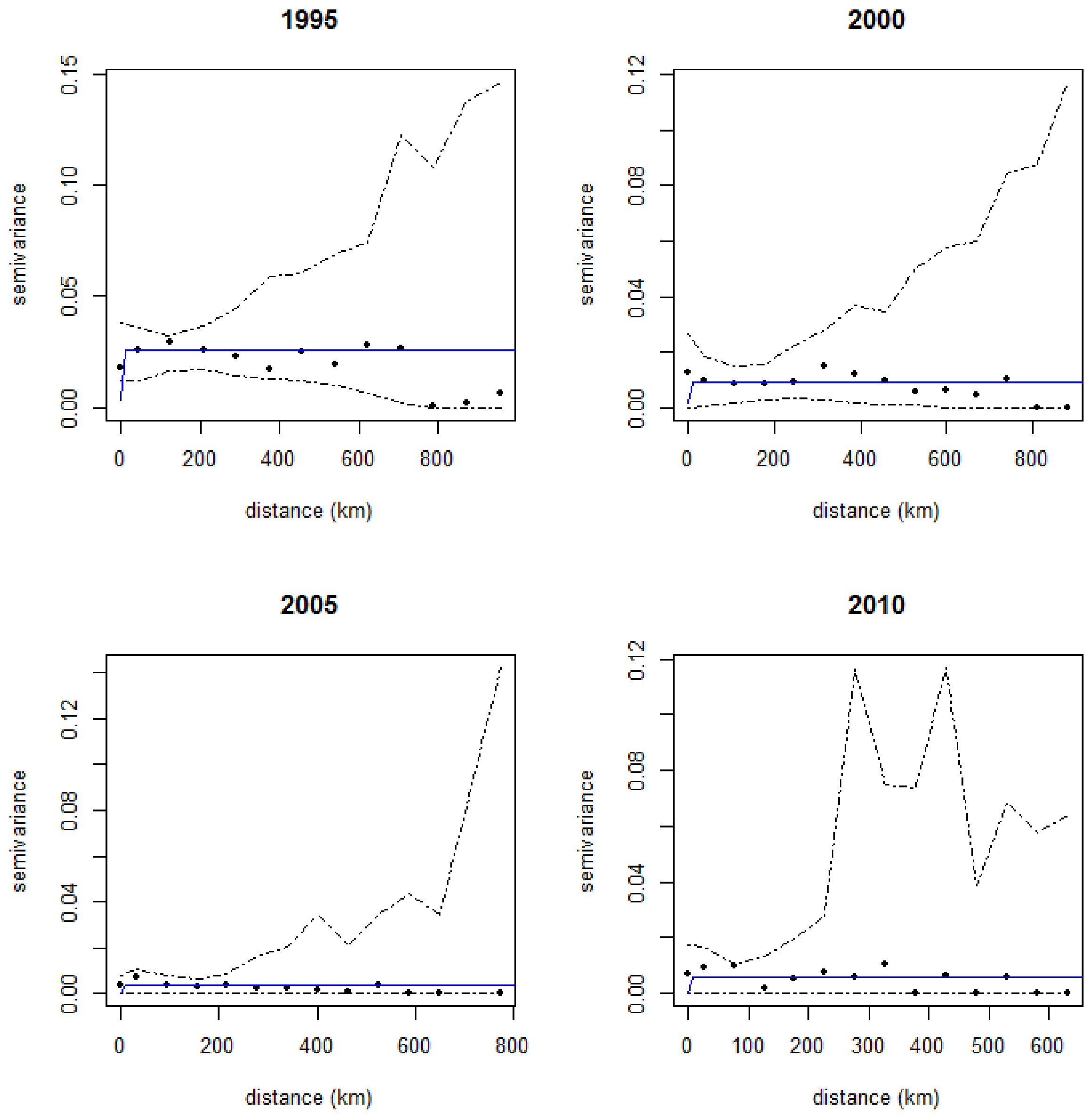

Supplementary Figure 3. Semivariogram analysis of spatial trend in the FoI for the years 1995, 2000, 2005 and 2010. The black circles represent the mean semivariance values between pairs of locations with a separation distance. The red solid line is the fitted theoretical variogram model illustrated with an exponential function. (Results were no different when other functions such as the Gaussian and spherical models were tested.) The dot-dashed lines represent the limits (95% non-parametric CI) of the envelope of variance expected by chance, obtained by randomly reassigning the values of force-of-infection at data locations. As the empirical semivariance fell within their envelopes we can accept the null hypothesis of non-spatial autocorrelation.

Supplementary Table 1. Characteristics of the serosurveys included in the analyses

Survey number	Source [Reference]	Type of source	Subnational level (first administrative level)	Year of survey	Test(s) used	Sample size	Observed overall prevalence	Type of setting	Age (years)	
									Min	Max
1	Orozco Vargas et al., 1998 [7]	Paper	Boyacá	1998	ELISA	333	34%	rural	5	75
2	Orozco Vargas et al., 1998 [7]	Paper	Boyacá	1998	ELISA	356	5%	urban	5	75
3	Guhl et al. 2005 [9]	Paper & Report	Boyacá	2000	ELISA	2958	4%	mixed	2	16
4	MoH, 2015 [12]	Report	Boyacá	2007	ELISA	274	2%	rural	4	16
5	MoH, 2015 [12]	Report	Boyacá	2007	ELISA	277	1%	urban	5	16
6	MoH, 2015 [12]	Report	Boyacá	2007	ELISA	803	3%	rural	3	15
7	MoH., 2015 [12]	Report	Boyacá	2007	ELISA	92	2%	urban	5	15
8	MoH, 2015 [12]	Report	Boyacá	2007	ELISA	212	24%	indigenous	1	62
9	MoH, 2015 [12]	Report	Boyacá	2007	ELISA	689	6%	rural	2	60
10	MoH, 2015 [12]	Report	Boyacá	2007	ELISA	285	4%	urban	5	19
11	MoH, 2015 [12]	Report	Boyacá	2007	ELISA	590	2%	rural	4	17
12	MoH., 2015 [12]	Report	Boyacá	2007	ELISA	205	2%	urban	5	15
13	MoH, 2015 [12]	Report	Boyacá	2007	ELISA	124	1%	rural	4	15
14	MoH, 2015 [12]	Report	Boyacá	2007	ELISA	140	1%	rural	5	12
15	MoH, 2015 [12]	Report	Boyacá	2007	ELISA	166	4%	rural	2	14
16	MoH, 2015 [12]	Report	Boyacá	2007	ELISA	84	2%	urban	3	14
17	MoH, 2015 [12]	Report	Boyacá	2007	ELISA	508	1%	rural	5	15
18	MoH, 2015 [12]	Report	Boyacá	2007	ELISA	50	0%	urban	5	15
19	MoH, 2015 [12]	Report	Boyacá	2008	ELISA	323	2%	rural	2	17
20	MoH, 2015 [12]	Report	Boyacá	2008	ELISA	392	4%	urban	4	23
21	MoH, 2015 [12]	Report	Boyacá	2008	ELISA	484	2%	rural	3	54
22	MoH, 2015 [12]	Report	Boyacá	2008	ELISA	364	1%	urban	4	45

23	MoH, 2015 [12]	Report	Boyacá	2008	ELISA	121	1%	rural	5	17
24	MoH, 2015 [12]	Report	Boyacá	2008	ELISA	1680	1%	urban	5	20
25	MoH, 2015 [12]	Report	Boyacá	2008	ELISA	377	2%	mixed	1	19
26	MoH, 2015 [12]	Report	Boyacá	2008	ELISA	467	3%	rural	1	20
27	MoH, 2015 [12]	Report	Boyacá	2008	ELISA	81	4%	urban	1	24
28	MoH, 2015 [12]	Report	Boyacá	2009	ELISA	85	7%	indigenous	1	8
29	MoH, 2015 [12]	Report	Boyacá	2009	ELISA	79	3%	rural	1	6
30	MoH, 2015 [12]	Report	Boyacá	2009	ELISA	853	0%	rural	1	19
31	MoH, 2015 [12]	Report	Boyacá	2009	ELISA	1035	1%	urban	1	20
32	MoH, 2015 [12]	Report	Boyacá	2009	ELISA	200	2%	rural	1	49
33	MoH, 2015 [12]	Report	Boyacá	2009	ELISA	87	1%	urban	1	49
34	MoH, 2015 [12]	Report	Boyacá	2010	ELISA	154	1%	mixed	1	17
35	MoH, 2015 [12]	Report	Boyacá	2010	ELISA	561	1%	rural	1	18
36	MoH, 2015 [12]	Report	Boyacá	2010	ELISA	134	1%	urban	1	18
37	MoH, 2015 [12]	Report	Boyacá	2010	ELISA	76	0%	rural	1	17
38	MoH, 2015 [12]	Report	Boyacá	2010	ELISA	741	1%	urban	1	18
39	Cucunubá et al., 2012, 2013 [17,18]	Abstract & Report	Boyacá	2010	ELISA, IFAT, HAI	290	3%	rural	14	46
40	Cucunubá et al., 2012, 2013 [17,18]	Abstract & Report	Boyacá	2010	ELISA, IFAT, HAI	148	3%	urban	14	43
41	MoH, 2015 [12]	Report	Boyacá	2011	ELISA	760	1%	rural	1	21
42	MoH, 2015 [12]	Report	Boyacá	2011	ELISA	311	2%	urban	1	20
43	MoH, 2015 [12]	Report	Boyacá	2011	ELISA	158	0%	rural	1	20
44	MoH, 2015 [12]	Report	Boyacá	2011	ELISA	311	0%	urban	1	19
45	Angulo-Silva, 2001 [8]	CINTROP/ UIS Report	Santander	1998	ELISA	926	29%	rural	1	98
46	Angulo-Silva, 2001 [8]	Papers & CINTROP/ UIS Report	Santander	2000	ELISA	481	44%	rural	2	82
47	Guhl et al., 2005 [9]	Paper & Report	Santander	2000	ELISA	1802	7%	mixed	3	16
48	Castellanos-Dominguez et al., 2016 [23]	Paper	Santander	2011	ELISA, IFAT, HAI	788	5%	rural	14	45
49	Castellanos-Dominguez et al., 2016 [23]	Paper	Santander	2011	ELISA, IFAT, HAI	722	2%	urban	13	46

50	MoH, 2015 [26]	Report	Santander	2013	ELISA	354	0%	rural	1	53
51	MoH, 2015 [26]	Report	Santander	2013	ELISA	146	3%	urban	1	59
52	MoH, 2015 [26]	Report	Santander	2014	ELISA	1152	0%	mixed	1	21
53	MoH, 2015 [26]	Report	Santander	2014	ELISA	454	0%	mixed	1	67
54	MoH, 2015 [26]	Report	Santander	2014	ELISA	573	0%	rural	1	20
55	MoH, 2015 [26]	Report	Santander	2014	ELISA	162	0%	urban	2	19
56	MoH, 2015 [26]	Report	Santander	2013	ELISA	291	0%	mixed	4	19
57	MoH, 2015 [26]	Report	Santander	2013	ELISA	390	1%	rural	1	28
58	MoH, 2015 [26]	Report	Santander	2013	ELISA	80	0%	urban	1	18
59	MoH, 2015 [26]	Report	Santander	2014	ELISA	351	0%	rural	1	18
60	MoH, 2015 [26]	Report	Santander	2014	ELISA	103	0%	urban	1	43
61	MoH, 2015 [26]	Report	Santander	2013	ELISA	523	0%	rural	1	66
62	MoH, 2015 [26]	Report	Santander	2013	ELISA	330	0%	urban	1	49
63	MoH, 2015 [26]	Report	Santander	2014	ELISA	428	2%	rural	1	48
64	MoH, 2015 [26]	Report	Santander	2014	ELISA	775	0%	urban	1	46
65	MoH, 2015 [26]	Report	Santander	2013	ELISA	30	0%	mixed	4	13
66	MoH, 2015 [26]	Report	Santander	2013	ELISA	263	0%	rural	1	42
67	MoH, 2015 [26]	Report	Santander	2013	ELISA	457	1%	urban	1	33
68	MoH, 2015 [26]	Report	Santander	2014	ELISA	39	3%	mixed	5	37
69	MoH, 2015 [26]	Report	Santander	2014	ELISA	306	0%	mixed	1	38
70	MoH, 2015 [26]	Report	Santander	2014	ELISA	173	0%	urban	2	19
71	MoH, 2015 [26]	Report	Santander	2013	ELISA	1014	0%	rural	1	37
72	Dib et al., 2011 [13]	Report	SNSM	2007	ELISA	419	33%	indigenous	1	81
73	MoH, 2015 [26]	Report	Santander	2014	ELISA	61	0%	rural	5	15
74	Guhl et al., 2005 [9]	Paper & Report	Arauca	2000	ELISA	823	22%	mixed	2	16
75	Cucunubá et al., 2012, 2013 [17,18]	Abstract & Report	Arauca	2011	ELISA, IFAT, HAI	243	2%	rural	13	46
76	Cucunubá et al., 2012, 2013 [17,18]	Abstract & Report	Arauca	2011	ELISA, IFAT, HAI	379	2%	urban	12	45

77	OPS/PAHO, 2012 [24]	Report	Arauca	2012	ELISA, IFAT, HAI	304	49%	indigenous	3	70
78	Guhl et al., 2005 [9]	Paper & Report	Casanare	2000	ELISA	1137	10%	mixed	1	16
79	MoH, 2015 [11]	Report	Casanare	2005	ELISA, IFAT	411	2%	rural	9	9
80	Angulo-Silva, 2012 [15]	Report CINTROP/UIS	Casanare	2008	ELISA	492	12%	mixed	1	88
81	Bianchi et al., 2015 [16]	Paper	Casanare	2009	ELISA, IFAT, HAI	1338	4%	rural	5	18
82	Bianchi et al., 2015 [16]	Paper	Casanare	2009	ELISA, IFAT, HAI	528	0%	urban	5	18
83	Cucunubá et al., 2012 [18,19]	Paper	Casanare	2010	ELISA, IFAT, HAI	280	7%	rural	13	43
84	Cucunubá et al., 2012 [18,19]	Paper	Casanare	2010	ELISA, IFAT, HAI	682	2%	urban	13	43
85	Gutierrez et al., 2013 [20]	Paper	Casanare	2010	ELISA, HAI	360	14%	urban	15	89
86	Gutierrez et al., 2013 [20]	Paper	Casanare	2010	ELISA, HAI	126	19%	rural	13	81
87	MoH, 2015 [11]	Report	Casanare	2011	ELISA, IFAT	1597	1%	rural	2	37
88	MoH, 2015 [11]	Report	Casanare	2011	ELISA, IFAT	1400	1%	rural	2	7
89	MoH, 2015 [11]	Report	Casanare	2013	ELISA, IFAT	926	0%	rural	2	17
90	MoH, 2015 [11]	Report	Casanare	2013	ELISA, IFAT	1215	1%	rural	2	22
91	Parra et al., 2004 [10]	Paper & Report	SNSM	2000	ELISA, IFAT	493	47%	indigenous	10	80
92	MoH, 2015 [26]	Report	Santander	2013	ELISA	419	1%	urban	1	60
93	Valencia-Hernández et al., 2015 [28]	Abstract & Report	SNSM	2014	ELISA or RDT	287	14%	indigenous	1	16
94	Guhl et al., 2005 [9]	Paper & Report	Antioquia	2000	ELISA	9800	0%	mixed	1	16
95	MoH, 2015 [21]	Report	Antioquia	2010	ELISA	66	6%	mixed	1	67
96	Guhl et al., 2005 [9]	Paper & Report	Bolivar	2000	ELISA	2253	0%	mixed	3	16
97	Cantillo-Barraza et al., 2014 [14]	Paper	Bolivar	2007	ELISA, IFAT	743	2%	rural	5	75
98	Cantillo-Barraza et al., 2015 [22]	Paper	Bolívar	2010	ELISA, IFAT	803	0%	rural	8	12
99	Guhl et al., 2005 [9]	Paper & Report	Cesar	2000	ELISA	1539	1%	mixed	1	16
100	Guhl et al., 2005 [9]	Paper & Report	Cundinamarca	2000	ELISA	2583	2%	mixed	1	16
101	Guhl et al., 2005 [9]	Paper & Report	Magdalena	2000	ELISA	7574	0%	mixed	1	16
102	Guhl et al., 2005 [9]	Paper & Report	Meta	2000	ELISA	1006	2%	mixed	2	16
103	Guhl et al., 2005 [9]	Paper & Report	Sucre	2000	ELISA	6040	0%	mixed	1	16

104	Guhl et al., 2005 [9]	Paper & Report	Tolima	2000	ELISA	2029	1%	mixed	2	16
105	Guhl et al., 2005 [9]	Paper & Report	Santander Norte	2000	ELISA	1188	6%	mixed	2	16
106	MSF, 2013 [25]	Report	Santander Norte	2012	ELISA	752	8%	indigenous	1	92
107	MSF, 2013 [25]	Report	Santander Norte	2012	ELISA	642	0%	urban	1	42
108	MSF, 2013 [25]	Report	Santander Norte	2012	ELISA	858	0%	rural	1	60
109	MoH, 2015 [27]	Report	Guainía	2013	ELISA	365	1%	rural	1	95

Abbreviations: MoH, Ministry of Health; MSF, Médicos Sin Fronteras; CINTROP/UIS, Centro de Investigación en Enfermedades Tropicales, Universidad Industrial de Santander; OPS/PAHO, Organización Panamericana de la Salud/Pan American Health Organization; SNSM, Sierra Nevada de Santa Marta; ELISA, enzyme-linked immunosorbent assay; IFAT, immunofluorescence antibody test; HAI, hemagglutination inhibition assay; RDT, Rapid Diagnostic Test.

Supplementary Table 2. Deviance Information Criterion for selecting the best model fit for the force-of-infection of Chagas disease in Colombia in individual serosurveys

Survey number	DIC values							Best model		FoI per 1,000 at risk population per year			
	Constant FoI (Model 1)	Model with up to four discrete changes in FoI over time and five values of λ_i (Model 2)					Yearly changes (Model 3)			Median	95% BCI		
		1	2	3	4	5		Model	DIC				
1	46	48	49	49	50	51	202	1	46	16.73	12.94	-	22.63
2	37	36	38	41	43	45	212	1	37	1.78	0.77	-	3.91
3	72	73	71	71	71	72	85	1	72	2.16	1.26	-	3.56
4	19	19	21	23	25	27	51	1	19	0.90	0.01	-	5.54
5	17	17	19	22	24	26	51	1	17	0.53	0.01	-	3.89
6	44	44	44	45	46	48	65	1	44	1.15	0.05	-	3.86
7	11	11	13	15	17	20	44	1	11	1.63	0.03	-	11.94
8	61	58	59	60	61	62	69	1	61	31.30	21.49	-	47.99
9	57	59	58	60	61	62	102	1	57	3.77	1.83	-	7.34
10	34	35	36	37	38	40	65	1	34	2.10	0.14	-	7.37
11	31	31	32	34	36	38	62	1	31	0.59	0.01	-	3.28
12	12	12	14	16	18	21	44	1	12	1.05	0.02	-	6.71
13	11	11	13	15	17	19	41	1	11	1.01	0.02	-	8.90
14	8	8	10	12	14	17	31	1	8	0.98	0.01	-	8.65
15	20	20	20	21	22	24	41	1	20	3.26	0.20	-	12.97
16	9	9	11	13	15	17	38	1	9	2.00	0.02	-	13.28
17	22	22	24	27	29	31	55	1	22	0.26	0.00	-	2.17
18	3	3	6	9	11	14	41	1	3	1.48	0.02	-	14.74
19	23	23	24	25	27	29	55	1	23	1.43	0.04	-	6.55
20	38	40	41	42	44	45	76	1	38	1.40	0.12	-	4.78
21	28	30	32	35	37	39	147	1	28	0.38	0.01	-	2.88
22	20	23	25	28	30	32	84	1	20	0.33	0.01	-	2.73
23	9	9	11	13	16	18	45	1	9	1.04	0.02	-	8.49
24	55	56	58	60	63	64	94	1	55	0.11	0.00	-	0.91
25	30	30	30	32	34	35	63	1	30	0.84	0.03	-	3.78
26	42	43	42	43	44	46	72	1	42	1.86	0.14	-	6.05
27	15	18	20	22	24	26	62	1	15	4.49	0.13	-	22.53
28	18	18	20	21	23	25	29	1	18	18.97	2.07	-	65.52
29	10	10	11	13	14	14	18	1	10	7.96	0.13	-	44.10
30	36	36	38	41	44	46	81	1	36	0.11	0.00	-	1.07
31	40	40	42	45	47	50	85	1	40	0.11	0.00	-	0.96
32	18	21	23	26	27	30	131	1	18	0.96	0.02	-	7.05
33	7	10	13	16	19	24	57	1	7	1.72	0.02	-	15.77
34	12	12	14	16	17	20	48	1	12	0.88	0.02	-	7.22
35	29	29	31	33	36	38	69	1	29	0.25	0.00	-	2.29
36	11	11	13	15	17	19	50	1	11	1.23	0.03	-	10.28

37	4	4	7	10	12	15	45	1	4	1.14	0.02	-	12.47
38	33	33	35	38	40	43	73	1	33	0.20	0.00	-	1.75
39	42	41	42	43	45	46	122	1	42	0.20	0.00	-	1.75
40	26	26	27	29	31	32	118	1	26	1.56	0.43	-	5.43
41	42	42	44	46	49	51	88	1	42	0.16	0.00	-	1.45
42	26	26	27	29	30	32	65	1	26	0.84	0.02	-	4.79
43	8	8	10	13	16	18	56	1	8	0.48	0.01	-	4.80
44	18	18	20	23	25	28	65	1	18	0.21	0.00	-	2.09
45	279	277	278	279	279	279	406	1	279	17.01	14.37	-	21.13
46	248	236	234	234	233	234	349	2[b]	236	10.80	4.28	-	19.78
47	65	66	66	67	67	67	71	1	65	6.24	4.57	-	8.86
48	87	83	84	84	85	86	135	1	87	2.45	1.62	-	3.89
49	58	57	57	58	60	61	127	1	58	0.92	0.46	-	1.98
50	16	18	21	24	26	29	86	1	16	0.19	0.00	-	1.69
51	25	23	25	27	29	31	91	1	25	1.92	0.19	-	7.75
52	48	48	50	53	56	58	96	1	48	0.06	0.00	-	0.65
53	20	23	25	28	31	34	89	1	20	0.17	0.00	-	1.72
54	25	25	27	30	32	35	73	1	25	0.14	0.00	-	1.37
55	8	8	10	13	16	18	54	1	8	0.42	0.00	-	4.24
56	13	13	16	18	21	24	59	1	13	0.21	0.00	-	2.01
57	23	25	28	30	33	34	68	1	23	0.28	0.01	-	2.63
58	5	5	7	10	12	15	46	1	5	0.97	0.01	-	9.64
59	16	16	18	21	23	26	60	1	16	0.22	0.00	-	1.99
60	6	9	11	14	17	18	56	1	6	0.69	0.01	-	6.74
61	25	28	30	33	36	39	93	1	25	0.16	0.00	-	1.43
62	17	20	22	25	27	30	83	1	17	0.26	0.00	-	2.50
63	60	50	51	49	49	53	96	2[a]	50	0.26	0.00	-	2.29
64	40	40	42	44	47	49	114	1	40	0.12	0.00	-	1.11
65	3	3	5	8	10	13	35	1	3	0.12	0.00	-	1.11
66	13	17	20	23	26	29	67	1	13	0.26	0.00	-	2.42
67	22	25	27	30	33	35	87	1	22	0.18	0.00	-	1.65
68	7	11	12	13	14	16	38	1	7	3.52	0.08	-	27.64
69	14	17	19	22	24	26	62	1	14	0.26	0.00	-	2.70
70	8	8	11	14	16	19	55	1	8	0.43	0.01	-	4.32
71	40	43	45	48	50	53	92	1	40	0.09	0.00	-	0.90
72	24	30	33	36	39	40	78	1	24	0.20	0.00	-	1.92
73	4	4	6	9	12	14	35	1	4	1.65	0.03	-	16.35
74	68	67	59	60	60	61	67	1	68	27.91	22.93	-	35.60
75	32	32	33	34	36	37	119	1	32	1.44	0.49	-	4.05
76	33	34	34	35	37	38	123	1	33	0.92	0.32	-	2.51
77	31	47	29	29	29	29	34	1	31	35.60	22.46	-	60.95
78	76	72	73	73	73	73	83	1	76	9.76	7.33	-	13.67
79	5	5	5	6	6	8	14	1	5	2.89	1.16	-	7.16
80	173	161	163	165	167	169	299	2[a]	161	0.75	0.01	-	4.68
81	50	50	50	51	52	53	65	1	50	4.84	3.43	-	7.25
82	13	13	13	14	16	18	48	1	13	0.49	0.06	-	2.18
83	66	66	66	67	68	69	121	1	66	3.97	2.27	-	7.48
84	63	62	62	63	64	65	123	1	63	1.23	0.65	-	2.44

85	143	144	145	145	146	147	264	1	143	4.63	3.28	-	6.95
86	76	76	77	78	79	80	305	1	76	6.51	3.86	-	11.82
87	43	37	28	29	29	30	84	2[a]	37	1.22	0.59	-	2.65
88	13	13	11	13	15	18	21	1	13	2.91	1.13	-	7.24
89	4	4	6	8	10	12	42	1	4	0.16	0.00	-	1.10
90	15	15	16	17	19	20	53	1	15	0.99	0.45	-	2.24
91	153	153	153	153	154	155	185	1	153	32.14	26.80	-	41.09
92	179	175	175	174	173	174	240	1	179	31.38	24.61	-	42.62
93	62	62	63	64	65	66	78	1	62	19.40	12.27	-	32.63
94	370	373	376	378	381	383	410	1	370	0.01	0.00	-	0.09
95	11	13	14	15	18	19	83	1	11	2.92	0.10	-	16.58
96	89	91	94	97	99	102	128	1	89	0.04	0.00	-	0.34
97	28	27	28	28	29	30	183	1	28	0.65	0.31	-	1.40
98	5	5	6	7	9	11	27	1	5	0.39	0.03	-	1.71
99	46	49	51	54	56	58	82	1	46	0.08	0.00	-	0.78
100	46	47	49	50	52	54	73	1	46	0.25	0.00	-	1.38
101	219	221	224	226	229	232	257	1	219	0.01	0.00	-	0.12
102	30	32	34	36	37	39	61	1	30	0.36	0.01	-	2.13
103	238	240	243	245	248	251	277	1	238	0.01	0.00	-	0.13
104	55	57	60	62	65	67	91	1	55	0.06	0.00	-	0.61
105	50	51	50	50	51	51	65	1	50	4.57	2.78	-	7.65
106	210	154	157	160	162	165	276	2[a]	154	0.14	0.00	-	1.39
107	27	30	33	36	39	41	94	1	27	0.11	0.00	-	1.21
108	45	45	47	50	52	54	166	1	45	0.15	0.00	-	1.26
109	24	27	29	32	34	36	246	1	24	0.08	0.00	-	0.67

Abbreviations: DIC, Deviance Information Criterion (values correspond to the lowest DIC or to that of the most parsimonious model if the difference between DIC values is not greater than 10 units [29]); FoI, force-of-infection; BCI, Bayesian Credible Interval. Models 1–3 are described in the main text.

[a] 1 change in FoI.

[b] 2 changes in FoI.

Survey number as in Supplementary Table 1.

Supplementary Table 3. Deviance Information Criterion for selecting the best model fit for subnational-level trends in the force-of-infection of Chagas disease in Colombia

Subnational level (Department)	DIC values							Best model	Number of discrete changes
	Constant FoI (Model 1)	Model with up to four discrete changes in FoI over time and five values of λ_i (Model 2)					Yearly changes (Model 3)		
		1	2	3	4	5			
Antioquia	**382**	384	387	389	391	393	440	Model 1	0
Arauca	648	650	588	574	572	573	**419**	Model 3	Yearly
Bolívar	**136**	133	133	135	135	137	284	Model 1	0
Boyacá	1,678	**1,481**	1,483	1,482	1,482	1,481	1,558	Model 2	1
Casanare	931	891	884	879	885	883	**863**	Model 3	Yearly
Cesar	**46**	49	51	54	55	55	82	Model 1	0
Cundinamarca	**46**	47	49	50	52	54	73	Model 1	0
Guainía	**24**	27	29	32	34	37	246	Model 1	0
Magdalena	**219**	221	224	226	229	232	257	Model 1	0
Meta	**30**	32	34	36	37	39	61	Model 1	0
Santander Norte	402	**328**	330	331	333	335	394	Model 2	1
Santander	2,407	1,943	1,931	1,911	1,901	1,902	**1,544**	Model 3	Yearly
SNSM	**401**	402	404	405	404	403	428	Model 1	0
Sucre	**238**	240	243	245	248	250	277	Model 1	0
Tolima	**55**	57	60	62	65	67	91	Model 1	0

Abbreviations: DIC, Deviance Information Criterion (numbers in bold highlight the lowest DIC value or the value for the most parsimonious model if the difference between the DIC values is not greater than 10 units [29]); FoI, force-of-infection; SNSM, Sierra Nevada de Santa Marta. Models 1–3 are described in the main text.

Supplementary Table 4. Deviance Information Criterion for selecting the best model fit for setting-level trends in the force-of-infection of Chagas disease in Colombia in four different settings

Setting	DIC values							Best model	Number of discrete changes
	Constant FoI (Model 1)	Model with up to four discrete changes in FoI over time and five values of λ_i (Model 2)					Yearly changes (Model 3)		
		1	2	3	4	5			
Rural indigenous	**1,126**	1,128	1,130	1,130	1,131	1,133	1,144	Model 1	0
Rural (non-indigenous)	3,291	2,972	2,955	2,933	2,919	2,912	**2,758**	Model 3	Yearly
Urban	1,057	927	**912**	907	906	910	1,060	Model 2	2
Mixed	2,834	**2,676**	2,678	2,680	2,681	2683	2,765	Model 2	1

Abbreviations: DIC, Deviance Information Criterion (numbers in bold highlight the lowest DIC value or the value for the most parsimonious model if the difference between the DIC values is not greater than 10 units [29]); FoI, Force-of-infection. Models 1–3 are described in the main text.

Supplementary References

1 Afonso AM, Ebell MH, Tarleton RL. A systematic review of high quality diagnostic tests for Chagas disease. *PLoS Negl Trop Dis* 2012;**6**:e1881. doi:10.1371/journal.pntd.0001881

2 McV. Messam LL, Branscum AJ, Collins MT, *et al.* Frequentist and Bayesian approaches to prevalence estimation using examples from Johne's disease. *Anim Heal Res Rev* 2008;**9**:1–23. doi:10.1017/S1466252307001314

3 Rudge JW, Webster JP, Lu D-B, *et al.* Identifying host species driving transmission of schistosomiasis japonica, a multihost parasite system, in China. *Proc Natl Acad Sci U S A* 2013;**110**:11457–62. doi:10.1073/pnas.1221509110

4 Gordis L. Assessing the Validity and Reliability of Diagnostic and Screening Tests. In: Elsevier Saunders, ed. *Epidemiology*. Philadelphia: 2004. 88–115.

5 Diggle PJ, Tawn JA, Moyeed RA. Model-based geostatistics. *J R Stat Soc Ser C (Applied Stat* 2002;**47**:299–350. doi:10.1111/1467-9876.00113

6 Mazzella A, Mazzella A. The Importance of the Model Choice for Experimental Semivariogram Modeling and Its Consequence in Evaluation Process. *J Eng* 2013;**2013**:1–10. doi:10.1155/2013/960105

7 Orozco LC, Camargo D, Gualdrón LE, *et al.* Seroprevalencia de infección por Trypanosoma cruzi en población urbana y rural de Guateque, Colombia. *Rev Fac Med UN Col* 1998;**48**:139–42.

8 Angulo-Silva VM. Ensayo de Estrategias de Control de la Transmisión Vectorial de la Enfermedad de Chagas en Santander. Informe Final CINTROP/UIS, OMS/TDR. Piedecuesta, Colombia: 2001.

9 Guhl F, Restrepo M, Angulo VM, *et al.* Lessons from a national survey of Chagas disease transmission risk in Colombia. *Trends Parasitol* 2005;**21**:259–62. doi:10.1016/j.pt.2005.04.011

10 Parra GJ, Restrepo Isaza M, Restrepo BN, *et al.* Estudio de tripanosomiasis americana en dos poblados indígenas de la Sierra Nevada de Santa Marta. Colombia. *Rev CES* 2004;**18**:43–50.

11 Secretaría de Salud de Casanare. Registro de Encuestas Serológicas para enfermedad de Chagas en el Departamento de Casanare. Informe ante el Ministerio de Salud y Protección Social. Yopal, Colombia: 2015.

12 Secretaría de Salud de Boyacá. Registro de Encuestas Serológicas para enfermedad de Chagas en el Departamento de Boyacá. Informe ante el Ministerio de Salud y Protección Social. Tunja, Colombia: 2015.

13 Dib JC. *Enfermedad de Chagas en las comunidades indígenas de la Sierra Nevada*

de Santa Marta. Bogota, Colombia: : Organizacion Panamericana de la Salud 2011.
14 Cantillo-Barraza O, Chaverra D, Marcet P, *et al.* Trypanosoma cruzi transmission in a Colombian Caribbean region suggests that secondary vectors play an important epidemiological role. *Parasit Vectors* 2014;**7**:381. doi:10.1186/1756-3305-7-381
15 Angulo-Silva VM. Rol de las poblaciones de Rhodnius prolixus de palmas en la transmisión de Trypanosoma cruzi en zonas intervenidas en los Llanos Orientales de Colombia. Informe Final CINTROP/UIS Colciencias. Bucaramanga, Colombia: 2012.
16 Bianchi F, Cucunubá Z, Guhl F, *et al.* Follow-up of an Asymptomatic Chagas Disease Population of Children after Treatment with Nifurtimox (Lampit) in a Sylvatic Endemic Transmission Area of Colombia. *PLoS Negl Trop Dis* 2015;**9**. doi:10.1371/journal.pntd.0003465
17 Cucunuba Z, Valencia C, Flórez C, *et al.* Pilot program for surveillance of congenital Chagas disease in Colombia 2010-2011. Int. J. Infect. Dis. 2012;**16**:e343. doi:10.1016/j.ijid.2012.05.412
18 Cucunubá ZM, Puerta CJ, Floréz AC, *et al.* Informe técnico final ante Colciencias. Proyecto “Desarrollo e implementación de un programa piloto de vigilancia de Chagas congénito en Colombia. Bogotá, Colombia: 2013.
19 Cucunubá ZM, Flórez AC, Cárdenas A, *et al.* Prevalence and risk factors for Chagas disease in pregnant women in Casanare, Colombia. *Am J Trop Med Hyg* 2012;**87**:837–42. doi:10.4269/ajtmh.2012.12-0086
20 Gutierrez FRS, Trujillo Güiza ML, Escobar Martínez M del C. Prevalence of Trypanosoma cruzi infection among people aged 15 to 89 years inhabiting the Department of Casanare (Colombia). *PLoS Negl Trop Dis* 2013;**7**:e2113. doi:10.1371/journal.pntd.0002113
21 Secretaría de Salud de Antioquia. Registro de Encuestas Serológicas para enfermedad de Chagas en el Departamento de Antioquia. Informe ante el Ministerio de Salud y Protección Social. Medellín, Colombia: 2015.
22 Cantillo-Barraza O, Garcés E, Gómez-Palacio A, *et al.* Eco-epidemiological study of an endemic Chagas disease region in northern Colombia reveals the importance of Triatoma maculata (Hemiptera: Reduviidae), dogs and Didelphis marsupialis in Trypanosoma cruzi maintenance. *Parasit Vectors* 2015;**8**:482. doi:10.1186/s13071-015-1100-2
23 Castellanos-Domínguez YZ, Cucunubá ZM, Orozco LC, *et al.* Risk factors associated with Chagas disease in pregnant women in Santander, a highly endemic Colombian area. *Trop Med Int Health* 2016;**21**:140–8. doi:10.1111/tmi.12634
24 Organización Panamericana de la Salud. Diagnóstico de situación de salud del pueblo Hitnú de Arauca. Informe ante el Ministerio de Salud y Protección Social.

Bogotá, Colombia: 2012.

25 Médicos Sin Fronteras. Haciendo Visible lo Invisible: Chagas en el Catatumbo, Norte de Santander, Colombia 2011-2012. Cúcuta: 2013.

26 Secretaría de Salud de Santander. Registro de Encuestas Serológicas para enfermedad de Chagas en el Departamento de Santander. Informe ante el Ministerio de Salud y Protección Social. Bucaramanga, Colombia: 2015.

27 Secretaría de Salud de Guainía. Registro de Encuestas Serológicas para enfermedad de Chagas en el Departamento de Guainía. Informe ante el Ministerio de Salud y Protección Social. Puerto Inírida, Colombia: 2015.

28 Valencia-Hernández CA, Caicedo A, Escribano D, *et al.* Pilot program for paediatric Chagas disease in remote indigenous communities in the Sierra Nevada de Santa Marta. In: *9th European Congress on Tropical Medicine and International Health*. Basel: 2015. A-744-0007-00331.

29 Spiegelhalter DJ, Best NG, Carlin BP, *et al.* Bayesian measures of model complexity and fit. *J R Stat Soc Ser B* 2002;**64**:583–639. doi:10.1111/1467-9868.00353
